# Efficacy of a Hip Brace for Hip Displacement in Children With Cerebral Palsy: A Randomized Clinical Trial

**DOI:** 10.1001/jamanetworkopen.2022.40383

**Published:** 2022-11-04

**Authors:** Bo Ryun Kim, Jin A. Yoon, Hyun Jung Han, Young Il Yoon, Jiwoon Lim, Seungeun Lee, Seon Cho, Yong Beom Shin, Hyun Jung Lee, Jee Hyun Suh, Joonyoung Jang, Jaewon Beom, Yulhyun Park, Jung-Hwa Choi, Ju Seok Ryu

**Affiliations:** 1 Department of Physical Medicine and Rehabilitation, Anam Hospital, Korea University College of Medicine, Seoul, South Korea; 2 Department of Rehabilitation Medicine, Pusan National University Hospital, Biomedical Research Institute, Pusan National University School of Medicine, Busan, South Korea; 3 Research Institute of Human Ecology, Chungbuk National University, Chungju-si, South Korea; 4 Chungbuk Technopark, Biocenter, Medical Device Health Team, Chungju-si, South Korea; 5 Department of Rehabilitation Medicine, Seoul National University Bundang Hospital, Seoul National University College of Medicine, Seongnam, South Korea; 6 Department of Rehabilitation Medicine, Jeju National University Hospital, Jeju National University College of Medicine, Jeju, South Korea; 7 Department of Rehabilitation Medicine, Ewha Women’s University medical center, Ewha Woman’s University School of Medicine, Seoul, South Korea; 8 SRC Rehabilitation Hospital, Gwangju-si, Gyeonggi-do, South Korea

## Abstract

**Importance:**

There is no consensus on interventions to slow the progress of hip displacement in patients with cerebral palsy.

**Objective:**

To investigate the efficacy of a novel hip brace in preventing progressive hip displacement in patients with cerebral palsy.

**Design, Setting, and Participants:**

This 2-group randomized clinical trial was conducted at 4 tertiary hospitals in South Korea from July 2019 to November 2021. Participants included children aged 1 to 10 years with nonambulatory cerebral palsy (Gross Motor Function Classification System level IV or V). Block randomization was used to assign an equal number of patients to the study and control groups via computerized random allocation sequences. Data were analyzed from November to December 2021.

**Interventions:**

The intervention group wore the hip brace for at least 12 hours a day for the study duration (ie, 12 months). Follow-up evaluations were performed after 6 and 12 months of wearing the brace. Both groups proceeded with conventional rehabilitation therapy during the trial.

**Main Outcomes and Measures:**

The primary outcome was the Reimers migration index (MI) on radiography, as assessed by 3 blinded investigators. Primary outcome variables were analyzed using linear mixed models. Secondary outcomes include change in the Caregiver Priorities & Child Health Index of Life with Disabilities, on which lower scores indicate better quality of life.

**Results:**

A total of 66 patients were included, with 33 patients (mean [SD] age, 68.7 [31.6] months; 25 [75.8%] boys) randomized to the intervention group and 33 patients (mean [SD] age, 60.7 [24.9] months; 20 [60.6%] boys) randomized to the control group. The baseline mean (SD) MI was 37.4% (19.3%) in the intervention group and 30.6% (16.3%) in the control group. The mean difference of the MI between the intervention group and control group was −8.7 (95% CI, −10.2 to −7.1) percentage points at 6 months and −12.7 (95% CI, −14.7 to −10.7) percentage points at 12 months. The changes in the Caregiver Priorities & Child Health Index of Life with Disabilities were favorable in the study group and reached statistical significance at the 6-month follow-up compared with the control group (difference, −14.2; 95% CI, −25.2 to −3.3).

**Conclusions and Relevance:**

In this randomized clinical trial, the novel hip brace was significantly effective in preventing the progression of hip displacement, compared with the control group. It effectively improved quality of life in patients with nonambulatory cerebral palsy. Therefore, hip brace use could be a promising treatment method to delay hip surgery and improve the quality of life of patients with nonambulatory cerebral palsy.

**Trial Registration:**

ClinicalTrials.gov Identifier: NCT04033289

## Key Points

**Question:**

Can a newly designed hip brace prevent progressive hip displacement in children with nonambulatory cerebral palsy?

**Findings:**

In this prospective, single-blinded randomized clinical trial including 66 patients with nonambulatory cerebral palsy (Gross Motor Function Classification System level IV or V), the Reimers migration index at 12-month follow-ups was significantly decreased in the group wearing hip brace but significantly increased in the control group.

**Meaning:**

These findings suggest that this newly developed hip brace could be used more widely as a nonsurgical treatment option to prevent hip displacement in children with nonambulatory cerebral palsy.

## Teaser

This randomized clinical trial investigates the efficacy of a novel hip brace in preventing progressive hip displacement in children with nonambulatory cerebral palsy.

## Introduction

Many children with cerebral palsy present with various musculoskeletal deformities associated with poor biomechanical alignment during growth.^1,2,3^ Among them, progressive hip displacement (Reimers migration index (MI) >30%-33%) is the second most common musculoskeletal deformity.^4,5^ Gradual deterioration of MI is seen in these children, from 3.9% per year at Gross Motor Function Classification System (GMFCS) level VI to 9.5% at level V.^4,5,6,7,8^ Hip displacement may cause pain; fixed deformity; pelvic obliquity; scoliosis; loss of ability to sit, stand, and walk; and difficulty with dressing, bathing, and perineal care; these significantly impact function and quality of life (QOL).^9^

Several hip surveillance programs for the early identification of and intervention for children with hips at risk have been established to prevent hip displacement and the need for complex salvage surgery.^6,10,11,12,13^ However, there is conflicting evidence for conservative management to prevent hip displacement in patients with nonambulatory cerebral palsy.^9^ Therefore, effective treatment for hip displacement in children with cerebral palsy has mainly focused on surgery.^10,12,14^

Meanwhile, several studies regarding nonsurgical treatment for hip displacement, including various types of hip abduction braces, postural alignment seating systems, and botulinum toxin injection, have reported inconsistent results.^15,16,17,18,19,20^ In previous research, we showed that a seating system with medial knee support could act as a fulcrum, thereby accelerating progressive hip displacement in patients with nonambulatory spastic cerebral palsy.^21^ In a follow-up study, the electromyographic activity of adductor muscles was significantly decreased after introducing a dynamic hip compression bandage, suggesting potential benefits.^22^ Theoretically, hip compression bandages biomechanically stabilize and assist in the protective function of the ligament and capsule around the hip joints.

Our hypothesis is that a hip brace can stabilize the hip joints, reduce hip adductor activation, and assist the ligaments and capsule in protecting the hip joint, thereby decreasing the progression of hip displacement. Therefore, we aimed to investigate the efficacy of a newly designed hip brace in preventing progressive hip displacement in patients with nonambulatory cerebral palsy.

## Methods

### Study Design

This study was a multicenter, prospective, single-blinded randomized clinical trial, conducted from July 26, 2019, to November 30, 2021, at the rehabilitation units of 4 teaching hospitals in South Korea. The study protocol was approved by the institutional review boards of each hospital, and all methods were performed in accordance with relevant guidelines and regulations. The trial protocol and statistical analysis plan are shown in Supplement 1. All patients or their representatives provided written informed consent prior to participation. This study is reported following the Consolidated Standards of Reporting Trials (CONSORT) reporting guideline.

### Participants

The inclusion criteria for participation were (1) diagnosis of cerebral palsy, (2) age 1 to 10 years, (2) GMFCS^23^ levels IV or V, (3) quadriplegia or diplegia, and (4) written consent with permission of the child and caregiver. The exclusion criteria were patients who had undergone a hip joint surgery, were scheduled to receive surgery during the trial, or had received botulinum toxin injections in their hip muscles within the 3 months before the study commencement or within the study duration.

**Figure 1.**
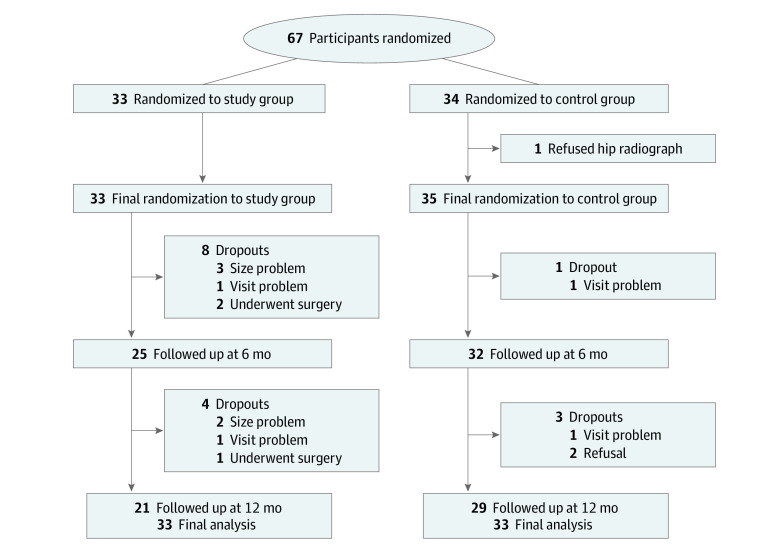
Flowchart of the Patients Through the Trial

Figure 1 shows the flow of participants through the trial. In this study, 1 participant refused to undergo the baseline evaluation after being assigned to the control group. Therefore, 66 participants (33 in the intervention group and 33 in the control group) were initially included. In the intervention group, 8 participants dropped out at the 6-month follow-up due to brace sizing issues (3 participants), visit problem (3 participants), and surgery (2 participants). At the 12-month follow-up, 4 more patients dropped out for the same reasons. In the control group, 1 participant dropped out due to a visit problem at the 6-month follow-up and 3 more participants dropped out due to visit problems (1 participant) and refusal (2 participants) at the 12-month follow-up (Figure 1).

### Randomization and Masking

Block randomization was used to randomly allocate participants in a 1:1 ratio to the intervention or control group via computerized random allocation sequences prepared by a statistician (random block size, 4). Eligible participants were randomly assigned immediately after baseline assessment. The randomization schedule and group allocation could only be accessed by the statistician and physical therapist (S.L.). Investigators involved in outcome assessment were blinded to the group allocation (B.R.K., J.A.Y., and J.L.).

### Intervention: Hip Brace

**Figure 2.**
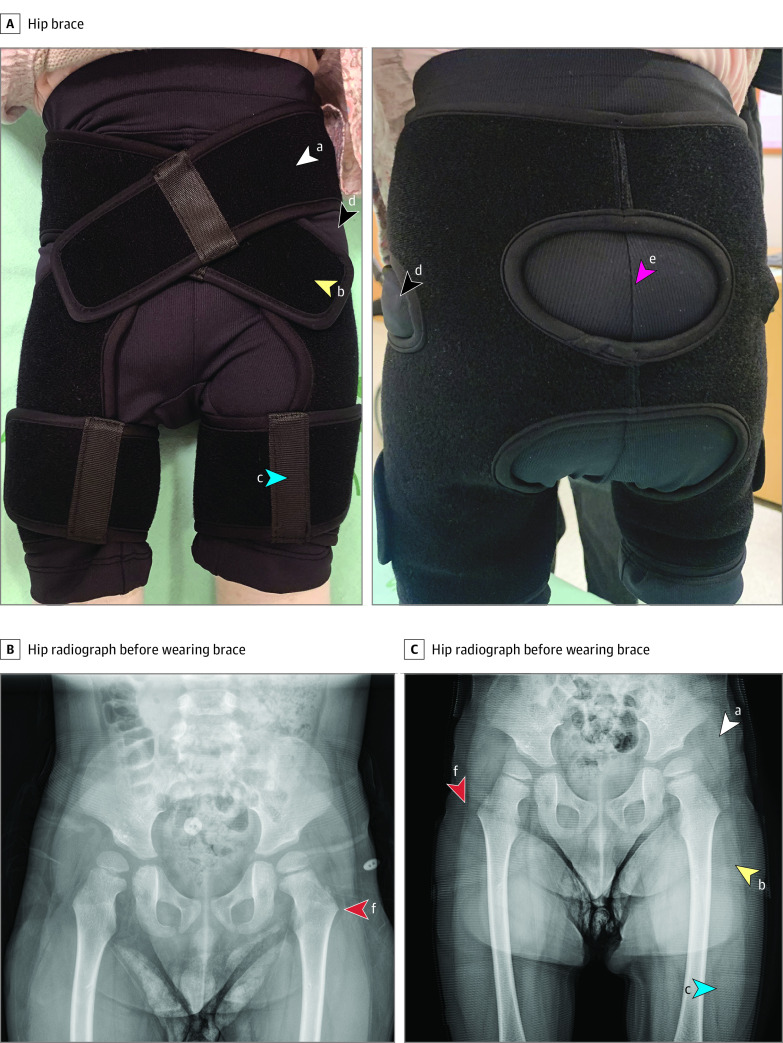
Hip Brace and Example Radiographs of a Patient Before and After Wearing the Hip Brace ^a^Upper straps were designed to protect hip joints from displacement. ^b^Lower straps were designed to prevent coxa valga. ^c^Thigh straps were designed to prevent hip adduction. ^d^To maximize the preventive effect on hip joint displacement, the greater trochanter (d) should be located between the upper and lower straps. ^e^The round design was applied at the buttock area of the fabric to allow comfort when lying or sitting and to prevent movement of the orthosis. ^f^The hip brace compresses the capsule and ligaments around the hip joints where displacement occurs, thereby helping with normal alignment.

The hip brace was developed for research and approved by the South Korean Ministry of Food and Drug Safety as a Class 1 medical device. The brace is composed of inner pants and outer fabric bands. The inner pants and Velcro make the brace easy to put on and take off. The outer fabric bands comprise of 3 elements (upper, lower, and thigh straps). The upper straps were designed to protect hip joints from displacement, and the lower straps were designed to prevent coxa valga. The thigh straps were designed to prevent hip adduction (Figure 2A). To maximize the preventive effect on hip joint displacement, the greater trochanter should be located between the upper and lower straps. Figure 2B and C shows radiographs of the hip before and after wearing the brace. The hip brace compresses the capsule and ligaments around the hip joints where displacement occurs, thereby helping with alignment.

Using the hip brace prototype (based on the age 5 years), the mean value of the 3 measurements of clothing pressure in 7 pressure areas were evaluated. The clothing pressure measurement method was based on the European Committee for Standardization, 2001, and the pressure class is divided into 4 grades (I, II, III, and IV) according to the compression force. Pressure was measured using an air-injected clothing pressure sensor and measuring instrument TNL-AMI 3037 (AMI Techno) (Pressure Class III or higher can be applied as a medical device).^24^ The results showed compression pressure higher than level III class on upper, lower, and thigh straps (eFigure in Supplement 2).

### Procedure

After enrollment, all participants received clinical and radiographic evaluation. Clinical evaluation included hip abduction range of motion (ROM) at 0° and 90° hip flexion. For radiographic evaluations, total hip anterior posterior (AP), bilateral femur lateral, and whole-spine AP radiographs were obtained in the supine position, with bilateral hip and knee extension, without wearing the brace. MI was measured using picture archiving and communication system (PACS) image analysis. The MI is the percentage of the femoral head that lies outside the acetabulum.^21,25^ The radiographic evaluations were performed by 3 blinded examiners, and the mean values were used for our study.^25^ After randomization, the study group wore the hip brace for at least 12 hours a day during the study period (ie, 12 months). Follow-up evaluations were performed after 6 and 12 months of wearing the brace. Both groups proceeded with conventional rehabilitation therapy during the trial.

### Outcome Variables

The primary outcome was the MI at 12 months. The secondary outcomes included MI at 6 months, hip ROM at 0° and 90° hip flexion, Cobb angle, pain intensity, QOL of the patients and their caregivers, and their satisfaction scores for the brace. Cobb angle was measured to assess the effect of hip brace on scoliosis. A visual analog scale (VAS; range, 0-10, with 0 indicating no pain and 10, worst pain) was used to measure the pain intensity and the Caregiver Priorities & Child Health Index of Life with Disabilities (CPCHILD; lower values indicate better QOL) was used to evaluate the change in QOL.^26^ A Likert scale was used to assess the satisfaction scores for the hip brace (range, 1-5, with 1 indicating very satisfied and 5, very unsatisfied).^27^ Because patients with nonambulatory cerebral palsy may find it difficult to communicate, the VAS and CPCHILD were evaluated by asking the caregivers’ perceptions of what the patients were feeling.

### Statistical Analysis

The calculation of the sample size was based on a previous epidemiological study.^21^ In this study, the annual progression of MI in patients with nonambulatory cerebral palsy was measured to be a mean (SD) of 7.83% (8.73%) per year. Assuming that the MI decreased by 80% when using the hip brace, the MI of the study group was set to a mean (SD) of 1.57% (8.73%). With an α < .05 in the 2-tailed tests and a power of 80%, the target sample size of each group was 64 patients (32 in each group). Considering a dropout rate of 5%, the final sample size was determined to be a total of 68 patients.

Descriptive statistics were used to summarize participant characteristics using mean (SD) or number (percentage) as appropriate. Continuous data were assessed for skewness by visual inspection and using a normality test. Unadjusted mean (SD) values were computed for the primary and secondary continuous variables at baseline and 6 and 12 months of follow up.

There was excellent agreement for MI among the 3 outcome assessors (interclass correlation coefficient: 0.989). The *t* test and χ^2^ tests were used to examine the baseline group differences. Analyses of the primary outcome variables were undertaken using linear mixed models, as specified in the study protocol, with age, baseline MI, groups, and time as fixed variables and patients, center, and side as a random effect using an unstructured covariance structure. In the case of dropout due to surgery and other reasons, data after dropout were excluded from the analysis. For the intention-to-treat analyses, all available data at baseline and 6 and 12 months were used. Mean differences at 6 and 12 months were estimated by the group by time interaction term, with associated 95% CIs and *P* values. A negative mean difference was indicative of better outcome values in terms of the MI. As there were interactions between group and time factors (*P* < .05), the model including the interaction was used as the final model. All data were analyzed using R statistical software version 4.1.1 (R Project for Statistical Computing). A 2-sided 5% level of significance was used throughout the analyses. Data were analyzed from November to December 2021.

## Results

Table 1 shows the baseline characteristics of all participants. A total of 66 patients were included, with 33 patients (mean [SD] age, 68.7 [31.6] months; 25 [75.8%] boys) randomized to the intervention group and 33 patients (mean [SD] age, 60.7 [24.9] months; 20 [60.6%] boys) randomized to the control group. The mean (SD) baseline MI (mean value between the right and left sides) was 37.4 (19.3) in the intervention group and 30.6 (16.3) in the control group. There were no statistically significant differences in the demographic data, except the baseline MI between the groups.

**Table 1.** Demographic and Baseline Characteristics

Characteristic	Mean (SD)	
	Study (n = 33)	Control (n = 33)
Age, mo	68.7 (31.6)	60.7 (24.9)
Sex, No. (%)		
Boys	25 (75.8)	20 (60.6)
Girls	8 (24.2)	13 (39.4)
Height, cm	103.7 (11.6)	101.2 (14.6)
Body weight, kg	15.4 (3.4)	16.3 (6.6)
GMFCS level, No. (%)		
IV	15 (45.5)	15 (45.5)
V	18 (54.5)	18 (54.5)
Migration Index, %		
Right	40.6 (20.0)	29.2 (13.6)
Left	34.3 (18.2)	32.0 (18.6)
Bilateral	37.4 (19.3)	30.6 (16.3)
Physical therapy time, min	179.8 (91.7)	202.5 (165.8)
Occupational therapy time, min	128.0 (65.1)	119.3 (69.0)
Interval from screening to 6-mo follow-up, mo	192.2 (21.8)	190.0 (21.1)
Interval from screening to 12-mo follow-up, mo	363.2 (23.6)	369.4 (18.4)
Hip abduction		
90° hip flexion	94.4 (39.2)	95.7 (36.6)
0° hip flexion	62.9 (25.8)	65.8 (27.4)
Knee flexion, median (IQR), °	130 (110-135)	135 (135-135)
Knee extension, median (IQR), °	20 (0-40)	0 (0-30)
Numeric Rating Scale pain score, median (IQR)	0 (0-4)	0 (0-4)
CPCHILD questionnaire score	37.6 (19.7)	33.9 (15.8)

Abbreviations: CPCHILD, Caregiver Priorities & Child Health Index of Life with Disability; GMFCS, Gross Motor Function Classification System.

**Figure 3.**
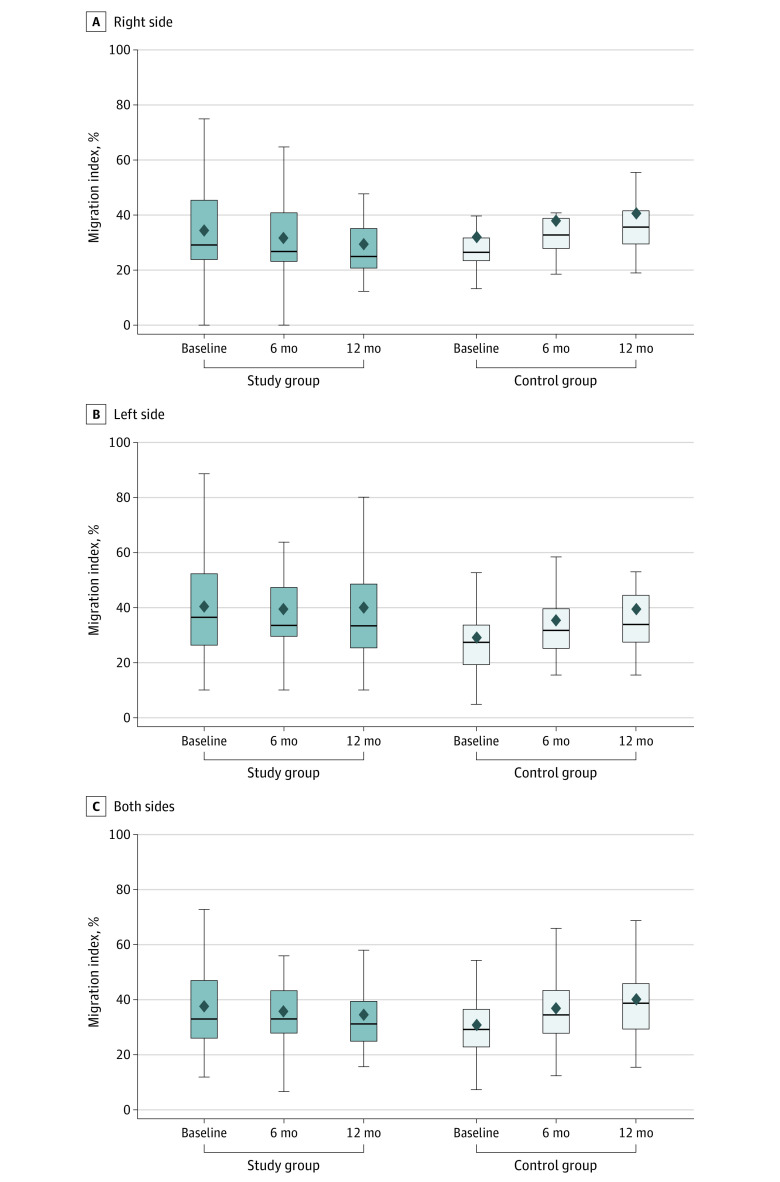
Mean Reimer Migration Index Scores at Baseline and 6 and 12 Months After 12 months, the migration index for both sides was significantly decreased from 37.4% to 34.6% in the intervention group and significantly increased from 30.6% to 40.1% in the control group (*P* < .001). Whiskers indicate range; top and bottom of the boxes, IQR values; dark line, median; diamond, mean.

The changes in the evaluated variables between groups are presented in Table 2 and Figure 3. The MI of the intervention group was significantly decreased by a mean (SD) −2.7 (6.9) percentage points at 6 months and −3.3 (6.9) percentage points at 12 months (mean [SD] annual progression rate: 6 months, −5.4 [13.8] percentage points; 12 months, −3.3 [6.9] percentage points; *P* < .001). However, the MI of the control group was significantly increased by a mean (SD) of 5.9 (7.4) percentage points at 6 months and 9.4 (10.9) percentage points at 12 months (mean [SD] annual progression rate: 6 months, 11.8 [14.8] percentage points; 12 months, 9.4 [10.9] percentage points; *P* < .001). The mean differences in the MI between groups was −8.7 (95% CI, −10.2 to −7.1) percentage points at 6 months and −12.7 (95% CI, −14.7 to −10.7) percentage points at 12 months.

**Table 2.** Comparisons of Changes in the Evaluated Variables Between Groups

Measure	Estimate, mean (SD)		*P* value	Estimation of difference (95% CI)
	Intervention (n = 33)	Control (n = 33)		
**Changes of MI, percentage points**				
6 mo				
Right	−3.2 (7.6)	6.2 (8.1)	<.001	−9.3 (−11.7 to −6.9)
Left	−2.3 (6.1)	5.7 (6.6)	<.001	−8.0 (−9.9 to −6.0)
Bilateral	−2.7 (6.9)	5.9 (7.4)	<.001	−8.7 (−10.2 to −7.1)
12 mo				
Right	−3.2 (7.3)	9.9 (11.7)	<.001	−13.1 (−16.2 to −10.1)
Left	−3.4 (6.6)	8.8 (10.0)	<.001	−12.2 (−14.9 to −9.5)
Bilateral	−3.3 (6.9)	9.4 (10.9)	<.001	−12.7 (−14.7 to −10.7)
**Changes of Hip ROM 90° hip flexion, °**				
6 mo	4.9 (28.6)	6.5 (26.7)	.84	−1.6 (−17.9 to 14.7)
12 mo	9.6 (35.6)	0.9 (25.2)	.33	8.7 (−8.9 to 26.3)
**Changes of Hip ROM 0° hip flexion, °**				
6 mo	0.8 (25.4)	−0.5 (19.7)	.84	1.4 (−11.8 to 14.5)
12 mo	9.7 (28.2)	6.8 (25.7)	.71	2.9 (−12.9 to 18.7)
**Changes of Cobb angle, °**				
6 mo	−0.2 (1.4)	1.1 (1.2)	.48	−1.3 (−5.1 to 2.4)
12 mo	−0.3 (1.2)	−0.6 (0.8)	.83	0.3 (−2.4 to 3.0)
**Changes of pain, median (IQR), points**				
6 mo	0 (−1 to 0)	0 (−0.25 to 0)	.38	−0.6 (−2.1 to 0.8)
12 mo	0 (−1 to 1)	0 (0 to 1)	.70	−0.3 (−1.8 to 1.2)
**Satisfaction with hip brace, median (IQR), points**				
6 mo	3 (2 to 3)	NA	NA	NA
12 mo	3 (2 to 3)	NA	NA	NA
**Changes of CPCHILD**				
Total score				
6 mo	−3.5 (21.5)	10.7 (16.5)	.01	−14.2 (−25.2 to −3.3)
12 mo	3.2 (15.8)	10.8 (16.5)	.12	−7.6 (−17.2 to 1.9)
Subdomain score: Comfort and Emotions				
6 mo	−3.0 (5.7)	0.9 (5.7)	.02	−3.9 (−7.0 to −0.8)
12 mo	0.2 (4.0)	2.2 (5.2)	.15	−2.0 (−4.8 to 0.8)

Abbreviations: CPCHILD, Caregiver Priorities and Child Health Index of Life with Disability; MI, migration index; NA, not applicable; ROM, range of motion.

Based on a linear mixed model, the regression coefficient for the MI in the intervention group was −2.7 at 6 months and −3.7 at 12 months. In the control group, the MI regression coefficient was 5.8 at 6 months and 9.4 at 12 months (P < .001) (eTable in Supplement 2).

The CPCHILD was favorable in the intervention group and reached statistically significant levels at the 6-month follow up compared with the control group (difference, −14.2; 95% CI, −25.2 to −3.3; *P* = .01) (Table 2). Although the statistical significance of the CPCHILD disappeared, the change was still favorable for the study group at the 12-month follow-up (Table 2). Although not statistically significant, the overall pain score was more favorable for the study group at the 6- and 12-month follow-ups than for the control group (Table 2). The mean (SD) Likert scale score for satisfaction for the brace was 2.9 (0.8) at 6 months and 2.7 (0.8) at 12 months. Other clinical parameters, such as hip and knee ROM, were not significantly different between groups at the 6- and 12-month follow-ups (Table 2).

## Discussion

This randomized clinical trial found significant improvement of hip displacement in the intervention group compared with the control group. The results of this study are meaningful because the hip brace used in this study can provide significant treatment to delay surgery and improve quality of life, in relation to hip displacement in patients with nonambulatory cerebral palsy.

As risk factors of hip displacement, it is well established that hip displacement is more frequent in quadriplegia, presence of spasticity, GMFCS levels IV and V, anomalous femoral geometry (increased femoral anteversion and neck-shaft angle), and acetabular dysplasia.^4,7,13,28,29^ In the presence of hip adductor spasticity, hip displacement was shown to be aggravated nearly 2-fold by the hip abduction bar due to leverage effects and length tension.^21,22^ Biomechanically, increased adduction forces on the hip joint with an abduction bar are thought to create the torque on the femoral head, shifting it laterally out of the acetabulum.^21,22,30^ In previous studies, a direct relationship has been established between hip displacement and GMFCS levels. In other words, patients with nonambulatory cerebral palsy without spasticity also develop hip displacement.^4,13,14^ Since the ligaments and capsule, which act to protect hip joints in children, are very weak, they may loosen if forces continuously act to stretch these structures in various daily activities, and displacement of the hip joint occurs. If the displacement state persists, the condition is not reversed by pulvinar formation.^31^ The hip brace in this study was developed to reinforce the protection for the ligaments and capsule to prevent progression of hip displacement.

Interestingly, the displacement of the study group significantly improved. This may be because the laxity of the ligaments and capsule precedes the displacement, but the laxity is reversible, and the ligaments and capsule are tightened when compressed. Owing to this laxity, the MI changed every time plain radiographs were acquired and even improved after botulinum toxin injection.^16,32^ Therefore, hip braces can be used as a promising treatment method.

Until now, there has been no definite recommendation for hip brace to slow the progression of hip displacement.^9^ A hip abduction brace was used to prevent hip displacement.^33^ However, the origin of this brace started with an explanation of the concept and it was used in clinical practice without clear study.^34,35^ However, in previous studies, the hip abduction brace combined with botulinum toxin injection was shown to be ineffective in preventing hip displacement.^15,16,19^ These results may be attributed to the inclusion of patients with lower risk of displacement (GMFCS levels I-III), the selection of incidence of surgery as the primary outcome, ineffectiveness of the previous abduction brace, and botulinum toxin injection to hamstring rather than the hip adductors muscles.^15,16,19^ The spasticity of the adductor muscles at hip joints should be distinguished from the spasticity of the hamstring muscle at the knee joints, but it was not distinguished in previous studies.^15,16,19^ These studies^15,16,19^ used an unverified abduction brace together with botulinum toxin, which resulted in an erroneous conclusion that both were ineffective.

As multiple factors are associated with hip displacement, complex treatments targeting the diverse mechanisms of hip displacement can maximize the efficacy of hip protection and reduce complications or the need for surgery.^28,32,36^ Theoretically, we can apply weight-bearing exercises in a standing position to activate the hip abductors and stimulate the acetabulum. These methods might additionally be effective to prevent coxa valga, femoral anteversion, and acetabular dysplasia.^28,36^ To control adductor spasticity, we can apply botulinum toxin injection at the adductors or adductor tenotomy. A 2021 study^20^ reported that a botulinum toxin injection repeated at 6 months in these muscles significantly reduced muscle tone by 40% at 1, 2, 3, and 7 months, which remained below baseline levels at 12 months, and the progression of the hip MI was significantly lower than that of the control group. Furthermore, neurogenic denervation after repetitive botulinum toxin injection can result in permanent decrease in muscle contractility.^37^ Wearing a hip brace during ambulation or vibration therapy can maximize the efficacy of and minimize the complication rate due to excessive force at the hip joints.

Hip displacement and related surgery can significantly impact function and QOL in patients and their caregivers.^38,39^ However, after applying the brace, the patients in the intervention group had higher QOL scores at 6 months compared with the control group. Prevention of surgery can further relieve the burden on patients and caregivers. Based on these results, it is necessary to amend the research on hip displacement with cerebral palsy, which previously focused on surgical treatment, to new conservative treatments focused on prevention. In addition, further study with long-term follow up is needed to determine whether using a hip brace can delay surgery and improve quality of life.

### Limitations

This study has some limitations. First, the dropout rate was higher than expected. We started with the representative brace size for patients aged 3, 5, and 6 years, but some participants grew faster than expected and could not wear the brace. In addition, there were difficulties in patient enrollment and follow-up due to COVID-19. Although 3 patients dropped out after undergoing surgery, the MI values of these 3 patients did not change, and surgery was performed by the guardians’ decisions. Second, hip ROM and pain did not show a significant change. Meaningful outcomes may have been observed if a longer follow-up was performed. Third, during the block randomization, MI was not included as a factor; therefore, there were statistically significant differences in baseline data between the groups. Fourth, although it was recommended that the braces be worn for at least 12 hours every day, the actual wearing time was not measured.

## Conclusions

In this randomized clinical trial, the hip brace was effective in preventing hip displacement aggravation. It effectively slowed and improved displacement and improved QOL in patients with cerebral palsy. Therefore, brace use could comprise a promising treatment method to delay hip surgery in patients with cerebral palsy.

## Notes

Supplement 1.
Trial Protocol and Statistical Analysis Plan

Supplement 2. **eFigure. **Clothing Pressure Measurements **eTable.** Results of the Linear Mixed Model

Supplement 3.
Data Sharing Statement
