# Errors in Figures

**DOI:** 10.1001/jamanetworkopen.2022.47506

**Published:** 2022-12-01

In the Original Investigation titled “Efficacy of a Hip Brace for Hip Displacement in Children With Cerebral Palsy: A Randomized Clinical Trial,”^1^ published November 4, 2022, there were errors in the number of participants allocated to the control group in Figure 1 and the panel label for Figure 2C. This article has been corrected.^1^
