# Supplementary material for: Efficacy of a Hip Brace for Hip Displacement in Children With Cerebral Palsy: A Randomized Clinical Trial
Source: JAMA Netw Open. 2022 Nov 4;5(11):e2240383. doi: 10.1001/jamanetworkopen.2022.40383 (PMC9636519; doi:10.1001/jamanetworkopen.2022.40383)
Supplement: Supplement 1. — Trial Protocol and Statistical Analysis Plan [file jamanetwopen-e2240383-s001.pdf]

This supplement contains the following items:

1. Study Protocol

Version 1.2 (Page 2)

Version 1.13 (Page 23)

Summary of Changes from Version 1.2 to Version 1.13 (Page 52)

Study plan title (English)

The efficacy of hip protection device for hip dislocation in in patients with cerebral palsy in a prospective, double-blinded RCT

Version No: 2.0

Coordinating Investigator Affiliation: Department of Rehabilitation Medicine, Seoul National University Bundang Hospital

Coordinating Investigator Name: Ju Seok Ryu

PROTOCOL SYNOPSIS

Study title	The Clinical Efficacy of Hip Protection Orthosis for The Protection of Hip Dislocation in Patients with Severe Cerebral Palsy, Single Blinded, Open Label, Multicenter Clinical Trial
Coordinating Investigator	Ju Seok Ryu<br>Department of Rehabilitation Medicine, Associate Professor
Fund support institution	Korea Health Industry Development Institute

Study purpose	This study aims to verify the clinical effectiveness of a wearable medical device developed to prevent hip dislocation in children with severe cerebral palsy.
Study design	Prospective, Multi-center
Study period	IRB approval date ~ 2 years
Study subject	Children aged 1 to 10 years with spastic cerebral palsy of GMFCS IV and V
Number of study subjects	42 people (21 in the experimental group, 21 people in the control group)
Vulnerable research subjects	Pediatric group (all subjects)
Test medical device	Hip joint protection wearable medical device
Usage and Dosage	-
Method	- Patients diagnosed with severe cerebral palsy were included in the study.<br>- Randomization into experimental group and control group if consent was given to the study<br>- Radiologic and clinical evaluation were performed to evaluate hip dislocation.

	- The experimental group manufactured and applied hip-joint wearable medical devices, Continue existing rehabilitation treatment.<br>- The control group continued the existing rehabilitation treatment.<br>- 6 months and 1year, clinical evaluation and radiological evaluation were performed to determine the effectiveness.
Major Inclusion criteria	- Persons aged 1 to 10 years<br>- Children with severe cerebral palsy (GMFCS level IV, V)<br>- Patients who agreed to participate in the study
Major Exclusion criteria	- If you do not consent to the study<br>- Those who are judged to be difficult to participate in under the judgment of the research director<br>- If you have had hip joint surgery or plan to have surgery during clinical research<br>- Botulinum injections up to 3 months ago or are planning to undergo treatment during the clinical study period
Efficacy evaluation	1. Primary efficacy endpoint: hip dislocation through radiographic evaluation (%)<br>2. Secondary efficacy endpoints: degree of scoliosis through plain radiograph; Functional changes such as pain index, satisfaction with wearable medical devices for hip joint protection, hip and knee joint range of motion, discomfort when caring for a patient, and changes in the patient's overall quality of life were found.
Safety assessment	- Plain X -ray imaging and clinical evaluation; Since the medical device under study is also a first-class medical device in the form of pants, No risk.<br>- Report any possible side effects from time to time and conduct periodic monitoring.
Inspection/Visit Schedule	- Plain X-rays before the start of the study, Conduct clinical evaluation. Application of hip joint wearable protective medical device<br>- Thereafter, radiological evaluation and clinical evaluation were

	performed twice at 6 -month intervals.
Statistical analysis method	Clinical evaluation is statistically compared with the results of simple X-rays at the initial stage, 6 months later, and 1 year later (compare with the paired t-test or Wilcoxon signed rank test by looking at the normal distribution curve). In addition, the difference between the experimental group and the control group is verified by an independent t-test or Mann Whitney U test. In this case, the significance level is set to 0.05.
Expected effect and Expected results	Hip dislocation in patients with severe cerebral palsy is a very common complication that occurs in more than 80% of patients, but there is no treatment available to prevent it. In case of complications, there is no other treatment other than surgical treatment.<br>It is expected that this medical device will prevent hip dislocation, reduce complications in patients with severe cerebral palsy, improve quality of life, It is expected to reduce medical expenses.

Study plan

1. Title: The Clinical Efficacy of Hip Protection Orthosis for The Protection of Hip Dislocation in Patients with Severe Cerebral Palsy, Single Blinded, Open Label, Multicenter Clinical Trial

2. Name and address of the research institute

Seoul National University Bundang Hospital Rehabilitation Medicine
: 82, Gumi-ro 173beon-gil, Bundang-gu, Seongnam-si, Gyeonggi-do, 13620
Jeju National University Hospital Rehabilitation Medicine
: 15, Aran 13-gil, Jeju-si, Jeju-do, 63241

3. Name and title of the Coordinating Investigator and Sub-Investigator

A. Coordinating Investigator

Name	Affiliation	Major	Position	Contact
Ju Seok Ryu	Seoul National University Bundang Hospital	Department of Rehabilitation Medicine	Associate professor	031-787-7739

B. Sub-Investigator

Name	Affiliation	Major	Position	Contact
Joonyoung Jang	Seoul National University Bundang Hospital	Department of Rehabilitation Medicine	Fellow	010-3487-7667

C. Multi-center and Principal Investigator of the institutes

Name	Affiliation	Major	Position	Contact
Bo Ryun Kim	Jeju University Hospital	Department of Rehabilitation Medicine	Associate professor	010-9828-0610

D. Person in charge of research: Ah-reum Shin

E. Researcher: Ah-reum Shin, Seungeun Lee, Eun Gyeong Jang

F. Investigational device managers: Usually, the in-hospital medical device manager is in charge of the medical devices, but in this study, the measurements are made according to the patient and then provided, so the Areum Shin Research Institute is in charge.

4. Sponsor

1) Sponsor name and address: Not applicable.

2) Monitor personnel name and title: Not applicable.

5. Name and address of research fund support institution

: Korea Health Industry Development Institute, 187, Osongsaengmyeong 2-ro, Osong-eup, Heungdeok-gu, Cheongju-si, Chungcheongbuk-do, Republic of Korea

6. Expected study period

2 years after the date of IRB approval

7. Study target disease

Children with severe cerebral palsy (GMFCS level IV, V)

8. Background and purpose of the study

1) Research Background

(1) Definition of cerebral palsy

Cerebral palsy is a disease that causes abnormal posture and movement due to damage to the immature brain and is one of the most serious disorders occurring in children. Brain damage during pregnancy or before and after delivery is common, but various causes are combined. Brain damage is permanent but non-progressive, and movement disorders in children with cerebral palsy vary depending on the area and degree of brain damage. Cerebral palsy is often spastic, involuntary movement, ataxic, hypotonic type, It is classified as a mixed type, Among them, the rigid type accounts for about 70-80% . The incidence of cerebral palsy by the age of 12 years is 2-5 per 1000 population. The incidence rate is constant or slightly increasing.

(2) Hip dislocation problem in children with cerebral palsy

Children with cerebral palsy are caused by excessive stiffness of the muscles around the lower extremities, and the femur falls out posteriorly and outward. The timing of dislocation is very variable, and it can occur even at an early age of 2-3 years. In particular, the frequency of occurrence increases as standing or walking is delayed or lying down time increases.

clinically defined complex syndrome, not a pathological mechanism, the incidence of subluxation and dislocation is described in various ways, ranging from 3 to 59%, depending on the authors. The frequency of subluxation and complete dislocation of the hip joint in children with cerebral palsy It is the second most common musculoskeletal complication after deformity. In the case of subluxation and complete dislocation, surgical treatment is often required to correct the musculoskeletal system and continuous physical therapy is required after surgery.

Among children with cerebral palsy, the risk of dislocation increases by 3.9% for GMFCS IV and 9.5% for GMFCS V each year. Serial X-ray every 6 months is suggested as a guideline.

These hip joint problems adversely affect the walking ability of the patient because walking ability is an important part of quality of life in the future, children with cerebral palsy Research on therapeutic measures to reduce the risk of hip dislocation is needed.

(3) Conservative treatment in children with cerebral palsy

There is currently no confirmed conservative treatment method to prevent hip dislocation in children with cerebral palsy.

(4) radiological evaluation

Hip subluxation and dislocation can be evaluated by plain X-ray imaging. [Fig 2] Migration index, which measures the proportion of the femoral head not covered by the hip acetabulum, is widely used.

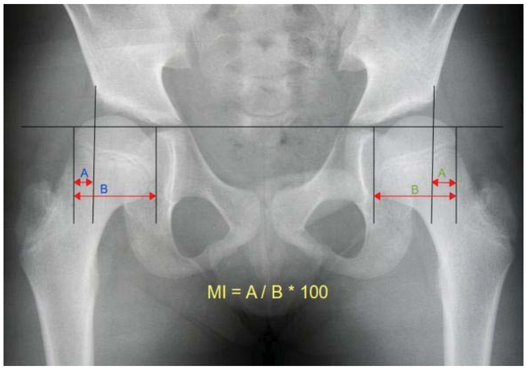

Figure 2. Hip migration index

2) Study hypothesis and purpose

hip dislocation occurs more frequently in children with cerebral palsy has not been clearly elucidated, it is expected that the force from strong contraction generated when the adductors are stiff will act in the direction of pulling out the femoral head, increasing the risk of hip dislocation in children with cerebral palsy. do. In addition, if the feet are crossed while lying down (scissoring), the risk of subluxation is rather reduced, and when the feet are in contact with each other, the principle of type 1 lever and the principle of length tension relationship work, and it is thought that the abduction torque of the hip joint will increase. At this time, the joint capsule surrounding the hip joint is stretched and ruptured, resulting in dislocation of the hip joint.

This researcher intends to develop a wearable auxiliary medical device to protect the joint capsule of the hip joint that surrounds the hip joint and apply it to prevent dislocation.

The hypothesis of this study is that the hip joint protection wearable auxiliary medical device has the effect of protecting the joint capsule and is expected to reduce dislocation.

To verify the above hypothesis, the purpose of this study is to prove the prevention of hip dislocation by applying this medical device to the study subjects.

hip subluxation and dislocation not only require surgical treatment and long-term

rehabilitation, but also adversely affect the child's walking and standing, the results of this study and subsequent clinical trials are expected to improve the quality of life of children with cerebral palsy. are expected to contribute.

9. Medical devices for clinical trials

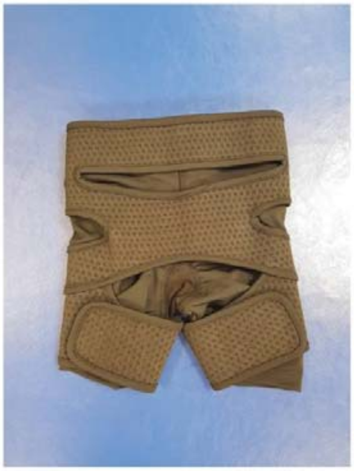

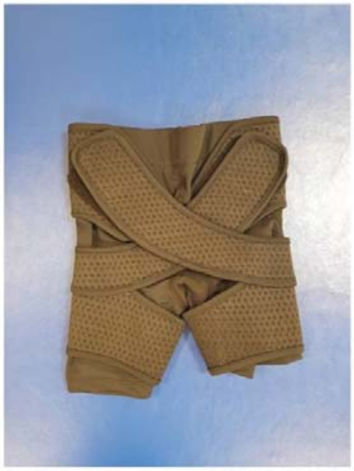

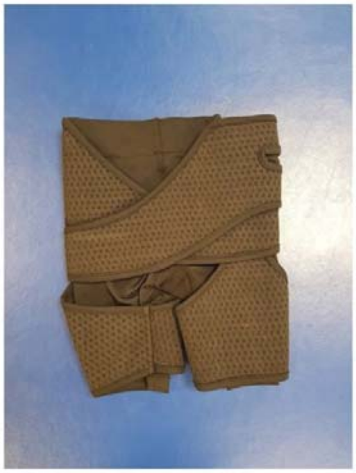

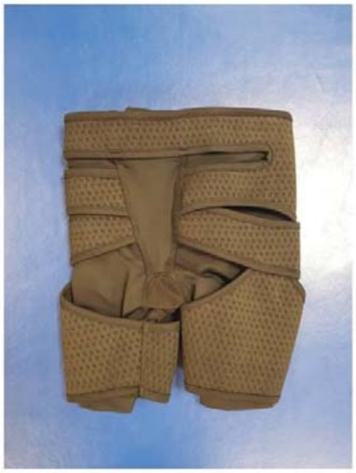

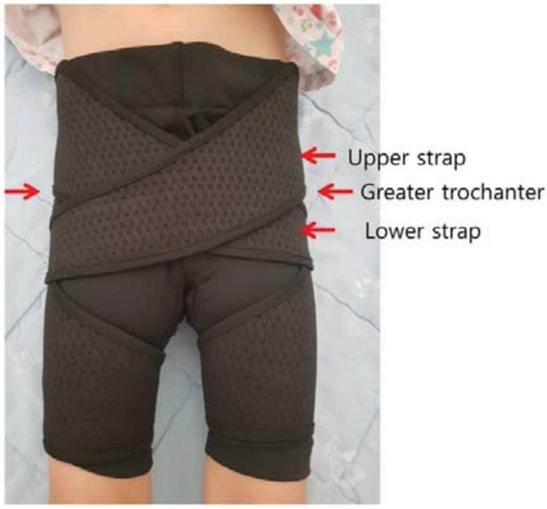

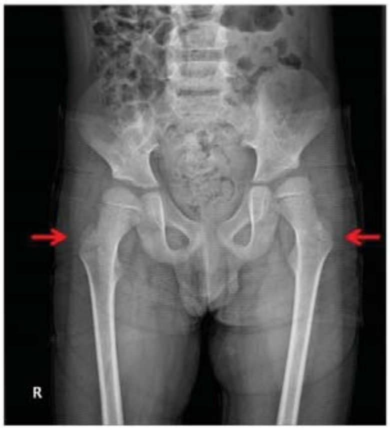

10. Selection criteria, exclusion criteria, target number of subjects and calculation basis

1) Inclusion criteria

- Over 1 year of age and under 10years of age
- Those with GMFCS level IV or V among children with severe cerebral palsy
- Those who have had quadriplegia or paraplegia for more than 6 months
- Hip subluxation /dislocation in children with spastic cerebral palsy occurred before the age of 1 year, so 56 children aged 1 to 10 years were selected. set.

2) Exclusion criteria

- If you do not consent to the study
- Those who are judged to be difficult to participate in under the judgment of the research director
- If you have had hip joint surgery or plan to have surgery during clinical research botulinum injections up to 3 months ago or are planning to undergo treatment during the clinical study period

3) Target number of subjects and calculation basis

- Previous study of this researcher, the MI of GMFCS IV and V patients before wearing auxiliary equipment was measured to be 26.89±17.10% (set as a control group) (APMR 2019, IS Kim, JS Ryu, etc.)

- This study, assuming that the MI decreased by 40% for GMFCS IV and 50% for GMFCS V when wearable auxiliary medical devices were worn, the MI of the experimental group was set to 16.134±17.1 and 13.445±17.1, respectively. se

- Study with Level of significance = 5%, Power = 80%, Type of test = two-sided subject Number Calculation When the program (G-power 3.1.9.2) is used, the total sample size is 11 people each in the experimental group and control group for GMFCS IV, and 8 people each in the experimental group and control group for GMFCS V. was calculated, and considering the dropout rate of 10%, a total of 42 experimental group control group to were planned to be recruited.

4) Study Subject Recruitment Plan

Patients with severe cerebral palsy who are undergoing treatment during the study participation period or children who have decided to participate in the study after reading the recruitment notice.

1) Specific research methods

Patients diagnosed with severe cerebral palsy were included in the study.

Assignment to experimental group and control group if consent to the study

Radiologic and clinical evaluation were performed to evaluate hip dislocation.

The experimental group manufactured and applied hip-joint wearable medical devices, Continue existing rehabilitation treatment.

The control group continued rehabilitation treatment. After 6 months and 1 year, clinical evaluation and radiological evaluation were performed to determine the effectiveness.

2) Control group setting and randomization method

To prevent an imbalance in the number of study subjects between the experimental group and the control group, a block randomization method is adopted.

Randomization was performed using Microsoft Excel program. 24 GMFCS IV subjects and 18 GMFCS V subjects were randomly assigned to two groups with a block size of 4 and an allocation ratio of 1:1. In the header of cells A1, B1, C1, and D1 in the Excel spreadsheet, enter Sequence, Random number, Block, and Group, respectively. ① Enter the numbers 1 to 24 and 1 to 18 in order in cell A, which is the sequence column, and assign a random function by entering the function = Rand() in B in the random number column. ③ In Block column C, repeat the numbers 1 to 6 and 1 to 4 in order. At this time, the numbers 1 to 4 or 6 are repeated 4 times, which means that 24 and 18 study subjects were assigned a block size of 4. ④ In the Group column, enter the experimental group and control group, respectively, followed by 12 and 9 people. Then, drag from B1 to D1 to select it, and select Home -> Sort & Filter -> Filter. When a scroll bar appears in each cell, click the scroll bar of C 1 to select ascending sort, and then click the scroll bar of C 1 to sort in ascending order to complete the randomization table (Kang, 2017). A random number table is created, and a new random function is generated at the start of the study. At this time, the random number on the sheet may change and the assignment may be changed, so copy or capture the value and keep it.

Multicenter Data Analysis Methods

Total number of study subjects is 42, and not only this institution but also Jeju National University Hospital recruits research subjects competitively. The randomization of study subjects is handled by the randomization officer of this institution, and when enrolling research subjects at each institution, randomization numbers are sequentially assigned to determine the experimental group and control group.

3) Drug administration/use, method, combination therapy, reasons for choosing the drug

None.

4) Observation items, clinical examination items, and observational examination methods

1. Primary efficacy endpoint: hip dislocation through radiographic evaluation (%)

2. Secondary efficacy endpoints: degree of scoliosis through plain radiograph ; pain index, functional changes such as satisfaction with wearable medical devices for hip joint protection, hip and knee joint range of motion , discomfort when caring for a patient, and changes in the patient's overall quality of life were investigated.

(1) hip dislocation test

Hip subluxation and dislocation can be evaluated by plain X-ray (Hip AP radiography). Migration index, which measures the proportion of the femoral head not covered by the hip acetabulum, is widely used. Measured at initial, 6 months, and 12 months.

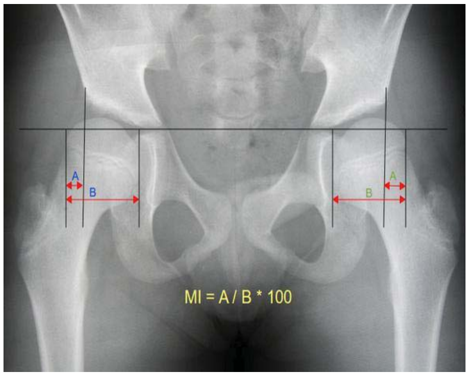

Hip migration index

(2) Whole spine AP

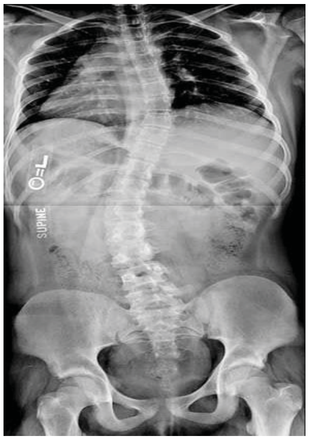

Examine the effect of hip fixation on scoliosis, the picture is taken while sitting in a sitting chair (chair used in radiology department) . The degree of scoliosis is measured by the Cobbs angle. Measured at initial , 6 months, and 12 months .

(2) Joint range of motion

Using a goniometer Measure the range of motion of the hip and knee of the patient

I. Hip abduction with hip at 90' flexion

II. Hip abduction with hip at 0' flexion

III. Hip adduction with hip at 0 ' flexion

IV. Hip flexion contracture (Thomas test)

V. Popliteal angle

(3) Questionnaire evaluation

I. Pain index (Visual analog scale) : In the case of children with cerebral palsy GMFCS IV and V, communication is difficult, Assess the patient's hip pain felt by the caregiver.

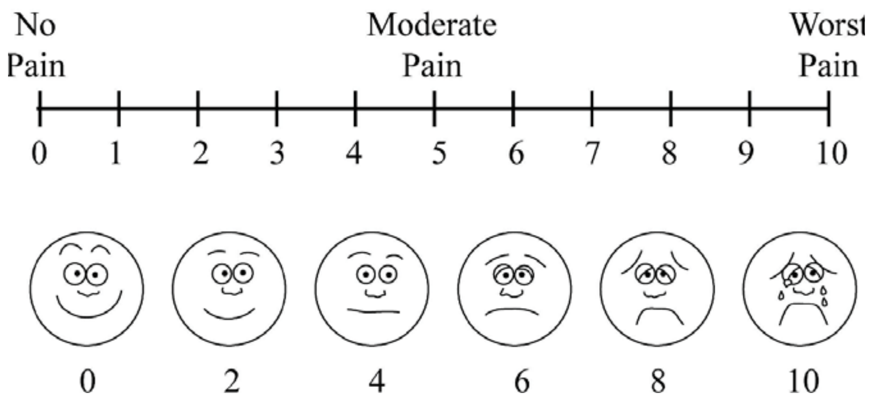

II. Likert scale

A. Evaluating the satisfaction of wearable medical devices for hip joint protection

B. Example of this study questionnaire)

Very satisfied - Satisfied - Moderate - Somewhat dissatisfied - Very dissatisfied

III. Child Health Index of Life with Disabilities (CPCHILD)

A. As a tool to evaluate the quality of life of patients with severe cerebral palsy It is a tool consisting of 9 items, evaluated by the caregiver. Each item is scored on a scale of 0 to 5, and each item is as follows.

B. daily activities, postures/movements, comfort and emotions, overall, of life quality, the child's of life vaginal Related of the item Investigate items related to hip contracture among CPCHILDs such as importance

C. example

5) From existing treatments and Study

To date, there is no established treatment method to prevent hip joint disease.

There are no medical devices to prevent hip dislocation.

6) **Benefit and Risk of Study Subjects**

(1) **Benefit of study subjects**

Current status of hip dislocation state can be examined through the trial, state of the and provide detailed training on how to manage it . receive Number there is

(2) Expenses for treatment of study subjects

There is no additional cost to be paid by the research subject as the researcher's cost for clinical evaluation, radiological examination, and orthosis is borne by the researcher.

(3) Forecast side effects/risks and countermeasures

Simple X -ray imaging is an examination method with proven safety. Also, in spastic cerebral palsy, periodic plain X-rays are recommended to evaluate the risk of dislocation. It does not increase the risk.

Because the hip dislocation assistive device is in the form of trousers, no special side effects are predicted, but if a side effect occurs, the cost of treatment is paid by the research fund.

7) Criteria for suspension and dropout

(1) Justice

Subjects who have completed the late evaluation according to the clinical trial protocol are considered to have completed the trial. Record whether or not all subjects participating in the clinical trial have completed the trial, and if the visit or observation is interrupted, the reason shall be stated.

(2) Criteria for stopping clinical trials

I. If the circumstances observed during the clinical trial make it unreasonable to proceed with the clinical trial, the principal investigator must request the clinical trial to be suspended to the clinical trial review committee can be stopped.

II. **Researcher** wishes to suspend a clinical trial for reasons such as safety of clinical trial medical devices, he/she may request to suspend the clinical trial to the Clinical Trial Review Committee and suspend the clinical trial according to the decision of the Clinical Trial Review Committee.

III. In case of temporary suspension for the treatment of adverse events that have occurred

IV. Suspension due to the occurrence of serious adverse events and abnormal medical device reactions

(3) Suspension of clinical trials

clinical trial stopped case The clinical trial director stopped up to the point proceeded All clinical trial related data in summary **The principal investigator** will keep it and discard it according to the procedure.

I. When a clinical trial is suspended, the clinical trial director must organize and deliver the case record, clinical trial progress, and results for the subjects who have progressed up to the time of the suspension to the **principal investigator** and return all test-related data (case records) to the **principal investigator**.

II. If the clinical trial is discontinued, the clinical trial director must immediately notify the subject of the discontinuation of the trial, ensure that appropriate measures and follow-up can be made, and notify the trial review committee in writing.

(4) The subject's leaving out standard

I. If the study is discontinued at the request of the subject or his/her representative

II. Clinical trial to the result Affect line Number there is surgery, drug or medical device in parallel used case

III. Clinical trial properly not done not case

IV. Inability to conduct early and late evaluations by the absence of the patient

V. Withdrawal of consent at the request of the subject or his/her representative

VI. In case tracking is not possible due to non-scheduled visit

VII. In case the subject dies due to reasons not related to the clinical trial

VIII. When the investigator determines that the suspension of the test is beneficial to the subject

IX. If the other investigator determines that there is a problem with the clinical trial

On the other hand, if the study is stopped due to safety problems due to the occurrence of adverse events or worsening of complications, the principal investigator shall take appropriate measures and follow -up until possible symptoms are recovered. Relevant information should be recorded in the case record.

⑤ Investigator dropout

I. If a subject is dropped out, the reason for dropping out and the data related to the clinical trial conducted before the dropout shall be recorded and kept.

II. Those who were dropped out are included in statistical processing through safety and efficacy evaluation unless there is a valid reason or justification.

III. For data obtained through clinical trials, a data set for efficacy analysis and safety analysis should be established.

8) Evaluation criteria for safety including side effects, evaluation methods and reporting methods

(1) Definition of Adverse Events

What is an 'Adverse Event (AE)'? clinical trial middle in the subject occurred every not intended not sign, laboratory Experiment of the result More than My back include), symptoms, or disease say, that clinical trials and must causal You don't have to have a relationship.

(2) Assessment of adverse events

I. severity assessment

More than case of moderate Grades 1-3 in 3 steps into Evaluate.

Grade	Severity	Evaluation standard
Grade 1	Mild	The degree to which the subject does not interfere with normal daily life (function) to a degree that is hardly felt. The extent to which most treatment is not required
Grade 2	Moderate	The degree to which the research subject may feel discomfort, and the degree to which normal daily life (function) is impaired. The

		degree to which the study subject may continue the trial but may require treatment
Grade 3	Severe	The degree to which the study subjects are very uncomfortable and daily life (function) is impossible, and continuous participation in the test is impossible. The extent to which treatment or hospitalization may be required

II. Evaluation of causal relationships with medical devices for clinical trials

More than case on manifestation clinical for testing with medical devices correlation Whether doctor in charge next and together Classify and test of the person in charge opinion add up

-Definitely related

The relationship between the occurrence of an adverse event and the use of a medical device for clinical trials is valid, and it is most likely explained by the use of the medical device for clinical trials than any other reason, and disappearance of an adverse event caused by the discontinuation of the medical device for clinical trials, and re-appearance of an adverse event caused by reusing the medical device. In addition, the symptom of adverse event is consistent with information already known about the medical device for the clinical trial or the medical device of the same class.

-Probably related

There is evidence that the relevant clinical trial medical device is used, the time sequence of the use of the clinical trial medical device and the occurrence of an adverse event is reasonable, and it is explained more probably by the use of the medical device for the clinical trial than other causes. When symptoms of abnormality caused by the discontinuation of the use of medical devices for testing disappear

-Relevance is suspected.

There is evidence that a clinical trial medical device has been used, and the time sequence of the use of the clinical trial medical device and the occurrence of an adverse

event is reasonable, and is judged to be due to the use of the clinical medical device at the same level as other possible causes. If the symptoms of anomalies caused by the discontinuation of the medical device for clinical trial disappear

-Probably not related

There is evidence that the medical device for the clinical trial is used, there is a more probable cause for the adverse event, and the symptoms of the adverse event caused by the discontinuation of the medical device for the clinical trial disappear or are ambiguous, and the reuse (only when reusable) result of the medical device for the clinical trial is adverse event or symptom is unclear or ambiguous

Definitely not related

-If the clinical trial medical device is not used, or the time sequence between the use of the clinical trial medical device and the occurrence of the adverse event is not valid or there are other obvious causes for the adverse event.

-Unable to evaluate (Unknown)

When information cannot be judged due to insufficient or conflicting information and cannot be supplemented or verified

(3) Evaluation criteria for safety

Adverse events, vital signs, laboratory test result should be considered for further evaluation

(4) How to evaluate safety

Vital signs and laboratory tests: For each evaluation, discomfort and skin problems are evaluated at each visit, and a safety evaluation questionnaire is filled out after the examination.

(5) How to report adverse events

I. Adverse case

Summary and analysis of the adverse case is for Treatment Emergent Adverse Events (TEAE). Application group not really Adverse events (AE), medical devices Adverse event (ADE),

serious Adverse events (SAE) About descriptive statistics (expression Number of subjects, incidence rate and manifestation number of cases) present between application groups Adverse events (AE), medical devices Adverse event (ADE), serious Adverse event (SAE) incidence. The difference whether Pearson's chi-square test or by Fisher's exact test Analyze.

Expressed every Adverse event (AE), medical devices Adverse events (ADEs) and serious Adverse events (SAE) about MedDRA latest version using System Organ Class (SOC) and Preferred Term (PT) according to to code coded Adverse case manifestation Number of subjects, incidence rate and number of occurrences My back by county present. Also serious in an abnormal case about details write

II. Serious adverse events

If serious abnormal case safety issues occur during follow up, over case of of Clinical Investigator or the test officer 24 hours within Korea Textile Research Institute report, and IRB.

9) **Criteria to evaluate the effect, evaluation and interpretation method (statistical analysis method, etc.)**

(1) Data analysis and statistical analysis method

1. Primary efficacy endpoint: hip dislocation through radiographic evaluation (%)

degree of scoliosis through plain radiograph; pain index, Functional changes such as satisfaction with wearable medical devices for hip joint protection, hip and knee joint range of motion, discomfort when caring for a patient, and changes in the patient's overall quality of life were investigated.

clinical evaluations were statistically compared (by Wilcoxon matched pairs signed rank test) as a result of plain X-rays at the initial stage and after 1 year. The difference in change values is verified by Mann Whitney U test between the experimental group and the control group.

P -value s is < 0.05, it is judged to be a significant level.

midway dropped out sleeping proper Reason or grounded Do not have one safety, efficacy evaluation through for statistical processing Including (ITT), all data are analyzed, and PP is analyzed by excluding dropouts and patients with missing data.

10) Treatment standards of study subjects after clinical trials

After clinical trial, the subject will follow the further treatment plan of the hospital, the subject should pay the further cost of the treatment. Otherwise, in case of side effects occurred during the trials, and the causality with medical devices is confirmed the principal investigator should offer the payment of the treatment.

In patients who drop out or had no reaction during the trial, the clinician should guide the subject to receive the appropriate treatment. The doctor should give instructions to re-visit the hospital at any time in case of unexpected delayed adverse effect.

15. References

1. Jeongrim Moon, Im Ji-eun, Young- Wan Moon, Song Dae-heon. Frequency of hip dislocation according to type and motion index in children with cerebral pals . Journal of Korean Rehabilitation Medicine 2004.

2. Park Joo-hyun; Kang Se- yoon , Yoon Yeon- joong , immature, A study on hip joint deformity in children with cerebral palsy . Journal of Korean Rehabilitation Medicine 1996.

3. Miller SD, Juricic M, Hesketh K, et al. Prevention of hip displacement in children with cerebral palsy: a systematic review. Dev Med Child Neurol. 2017.

4. Parrot J, Boyd RN, Dip PG, Dobson F: Hip displacement in spastic cerebral palsy: repeatability of radiologic measurement. J Ped Orthop 2002.

Study plan title

The clinical efficacy of hip protection orthosis for the protection of hip dislocation in patients with severe cerebral palsy, single blinded, randomized-control trial, multicenter clinical trial

Version No: 13.0

Coordinating Investigator Affiliation: Department of Rehabilitation Medicine, Seoul National University Bundang Hospital
Coordinating Investigator Name: Jaewon Beom

Study Overview

Study title	The clinical efficacy of hip protection orthosis for the protection of hip dislocation in patients with severe cerebral palsy, single blinded, randomized-control trial, multicenter clinical trial
Coordinating Investigator	Jaewon Beom<br>Department of Rehabilitation Medicine associate professor
Fund support institution	Korea Health Industry Development Institute

Study purpose	This study aims to verify the clinical effectiveness of a wearable medical device developed to prevent hip dislocation in children with severe cerebral palsy.

Study design	Prospective, Multi-center
Study period	IRB approval date ~ 2 years
Study subject	Children aged 1 to 10 years with spastic cerebral palsy of GMFCS IV and V
Number of study subjects	68 people (34 people in the experimental group, 34 people in the control group)
Vulnerable research subjects	Pediatric group (all subjects)
Test medical device	Hip joint protection wearable medical device
Usage and Dosage	
Method	- Patients diagnosed with severe cerebral palsy were included in the study.<br>- Randomization into experimental group and control group if consent was given to the study.<br>- Subject's body measurements were performed to measure the size of the hip joint wearable medical device.<br>- Radiologic and clinical evaluation were performed to evaluate hip dislocation.<br>- The experimental group manufactured and applied hip-joint wearable medical devices, Continue existing rehabilitation treatment.<br>- The control group continued the existing rehabilitation treatment.<br>- 6 months and 1year, clinical evaluation and radiological evaluation were performed to determine the effectiveness.
Major Inclusion criteria	- Persons aged 1 to 10 years<br>- Children with severe cerebral palsy (GMFCS level IV, V)<br>- Patients who agreed to participate in the study
Major Exclusion criteria	- If you do not consent to the study<br>- Those who are judged to be difficult to participate in under the

	judgment of the research director<br>- If you have had hip joint surgery or plan to have surgery during clinical research
Efficacy evaluation	1. Primary efficacy endpoint: hip dislocation through radiographic evaluation (%)<br>2. Secondary efficacy endpoints: degree of scoliosis through plain radiograph; Degree of femur anteversion through plain radiograph, pain index, Functional changes such as satisfaction with wearable medical devices for hip joint protection, hip and knee joint range of motion, discomfort when caring for a patient, and changes in the patient's overall quality of life were investigated.
Safety assessment	- Plain X -ray imaging and clinical evaluation; Since the medical device under study is also a first-class medical device in the form of pants, No risk.<br>- Report any possible side effects from time to time and conduct periodic monitoring.
Inspection/Visit Schedule	- Plain X-rays before the start of the study, Conduct clinical evaluation.<br>- Application of hip joint wearable protective medical device thereafter, radiological evaluation and clinical evaluation were performed twice at 6month interval .
Statistical analysis method	Statistical comparison of clinical evaluation results from simple X-rays at the initial stage and one year later (Compare with paired t-test or Wilcoxon signed rank test according to normality test). Also, statistically compare the difference between the initial, 6 months, and 1 year after simple X-ray results and change values for clinical evaluation. Repeatedly measured values are verified with a linear mixed model or a generalized estimating equation (GEE) between the experimental group and the control group. If the P-values are < 0.05, it is judged to be a significant level.
Expected effect	Hip dislocation in patients with severe cerebral palsy is a very common

and<br>Expected results	complication that occurs in more than 80% of patients, but there is no treatment available to prevent it. In case of complications, there is no other treatment other than surgical treatment.<br>It is expected that this medical device will prevent hip dislocation, Reduce complications in patients with severe cerebral palsy, improve quality of life, It is expected to reduce medical expenses.

Study Plan

1. **Study Title** : The Clinical Efficacy of Hip Protection Orthosis for The Protection of Hip Dislocation in Patients with Severe Cerebral Palsy, Single Blinded, Randomized-control trial, Multicenter Clinical Trial

2. Name and address of the research institute

Seoul National University Bundang Hospital Rehabilitation Medicine
: 82, Gumi-ro 173beon-gil, Bundang-gu, Seongnam-si, Gyeonggi-do, 13620
Jeju National University Hospital Rehabilitation Medicine
: 15, Aran 13-gil, Jeju-si, Jeju-do, 63241
Pusan National University Hospital Rehabilitation Medicine
: 179, Gudeok-ro , Seo-gu , Busan, 49241
Bundang Jesaeng Hospital Rehabilitation Medicine
: 20, Seohyeon-ro 180beon-gil, Bundang-gu, Seongnam-si, Gyeonggi-do, 13590
Korea University Hospital Rehabilitation Medicine
: 73 Goryeodae-ro, Seongbuk-gu, Seoul, 02841

3. Name and title of the Coordinating Investigator and Sub-Investigator

A. Coordinating Investigator

Name	Affiliation	Major	Position	Contact
Jaewon Beom	Seoul National University Bundang Hospital	Department of Rehabilitation Medicine	Associate professor	031-787-7739

B. Sub-Investigator

Name	Affiliation	Major	Position	Contact

Jiwoon Lim	Seoul National University Bundang Hospital	Department of Rehabilitation Medicine	Fellow	010-9699-6505

A. Multi-center and Principal Investigator of the institutes

Name	Affiliation	Major	Position	Contact
Hyun Jeong Lee	Jeju National University Hospital	Department of Rehabilitation Medicine	Medical professor	010-2743-3170
Jee Hyun Seo	Bundang Jesaeng Hospital	Department of Rehabilitation Medicine	Professor	010-6528-6578
Yong Beom Shin	Pusan National University Hospital	Department of Rehabilitation Medicine	Professor	051-240-7485
Bo Ryun Kim	Korea University Hospital	Department of Rehabilitation Medicine	Associate professor	010-9828-0610

B. Person in charge of research : Sun Cho Researcher

C. Investigational device managers: Usually, the in-hospital medical device manager is in charge of the medical device, but in this study, the measurements are made according to the patient and then provided, so the Seon Cho researcher is in charge.

4. Sponsor

1) Sponsor name and address: Not applicable.

2) Monitor name and title: Not applicable.

5. Name and address of research fund support institution

: Korea Health Industry Development Institute, 187, Osongsaengmyeong 2-ro, Osong-eup, Heungdeok-gu, Cheongju-si, Chungcheongbuk-do, Republic of Korea

6. Expected study period

2 years after the date of IRB approval

7. Study target disease

Children with severe cerebral palsy (GMFCS level IV, V)

8. Background and purpose of the study

1) Background

(5) cerebral palsy

Cerebral palsy is a disease that cause abnormal posture and movement due to damage to the immature brain and is one of the most serious disorders occurring in children. Brain damage during pregnancy or before and after birth is common, but in most case, various causes are combined, or the cause is unknown. Brain damage is permanent but non-progressive, and movement disorders in children with cerebral palsy vary depending on the location and extent of brain damage. Cerebral palsy is often classified into spastic type, involuntary movement type, ataxia type, hypotonic type, and mixed type, of which the spastic type accounts for about 70-80%. The frequency of cerebral palsy around the age of 12 years of age reaches 2 to 5 per 1,000 population, and the incidence rate is on a constant or slightly increasing trend.

(6) Hip dislocation problem in children with cerebral palsy

Hip dislocation in children with cerebral palsy is caused by excessive stiffness of the muscles around the lower extremities, and the femur falls out posteriorly and outward. The timing of dislocation is very diverse, and it can occur even at an early age of 2-3 years. In particular, the frequency of occurrence increases as standing or walking is delayed or lying down time increases.

Because cerebral palsy is a clinically defined complex syndrome, not a pathological mechanism, the incidence of subluxation and dislocation varies widely depending on the authors, ranging from 3 to 59%, but the frequency of hip subluxation and complete dislocation is cerebral palsy. It is the second most common musculoskeletal complication after equinus deformity in paralyzed children.

In the case of subluxation and complete dislocation, surgical treatment to correct the musculoskeletal is often required, and continuous physical therapy is required even after surgery.

Among children with cerebral palsy, the risk of dislocation increases by 3.9% for GMFCS IV and 9.5% for GMFCS V each year, dislocation examination through X-ray every 6 months is suggested as a guideline.

These hip joint problems adversely affect the walking ability of the patient because walking ability plays an important role in quality of life in the future, research on therapeutic measures to reduce the risk of hip dislocation is needed.

(7) Conservative treatment in children with cerebral palsy

There is currently no confirmed conservative treatment method to prevent hip dislocation in children with cerebral palsy.

(8) radiological evaluation

Hip subluxation and dislocation can be evaluated by plain X-ray imaging[Fig 2]. Migration index, which measures the proportion of the femoral head not covered by the hip acetabulum, is widely used.

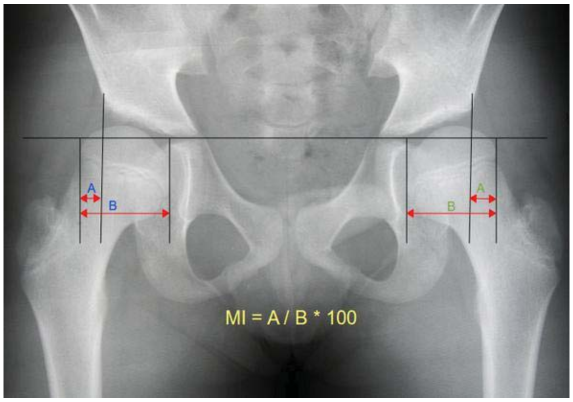

Figure 2 . Hip migration index

3) Hypothesis and purpose

Hip dislocation occurs more frequently in children with cerebral palsy has not been clearly elucidated, it is expected that the force from strong contraction generated when the adductors are stiff will act in the direction of pulling out the femoral head, increasing the risk of hip dislocation in children with cerebral palsy do. In addition, if the feet are crossed while lying down(scissoring), the risk of subluxation is rather reduced, and when the feet are in contact with each other, the principle of type 1 lever and the principle of length tension relationship work, and it is thought that the abduction torque of the hip joint will increase. At this time, the joint capsule surrounding the hip joint is stretched and ruptured, resulting in dislocation of the hip joint.

This researcher intends to develop a wearable auxiliary medical device to protect the joint capsule of the hip joint that surrounds the hip joint and apply it to prevent dislocation.

The hypothesis of this study is that the hip joint protection wearable auxiliary medical device has the effect of protecting the joint capsule and is expected to reduce dislocation.

To verify the above hypothesis, the purpose of this study is to prove the prevention of hip dislocation by applying this medical device to the study subjects.

hip subluxation and dislocation not only require surgical treatment and long-term rehabilitation, but also adversely affect the child's walking and standing, the results of this study and subsequent clinical trials are expected to improve the quality of life of children with cerebral palsy. are expected to contribute.

9. Medical devices for clinical trials

<1st prototype>

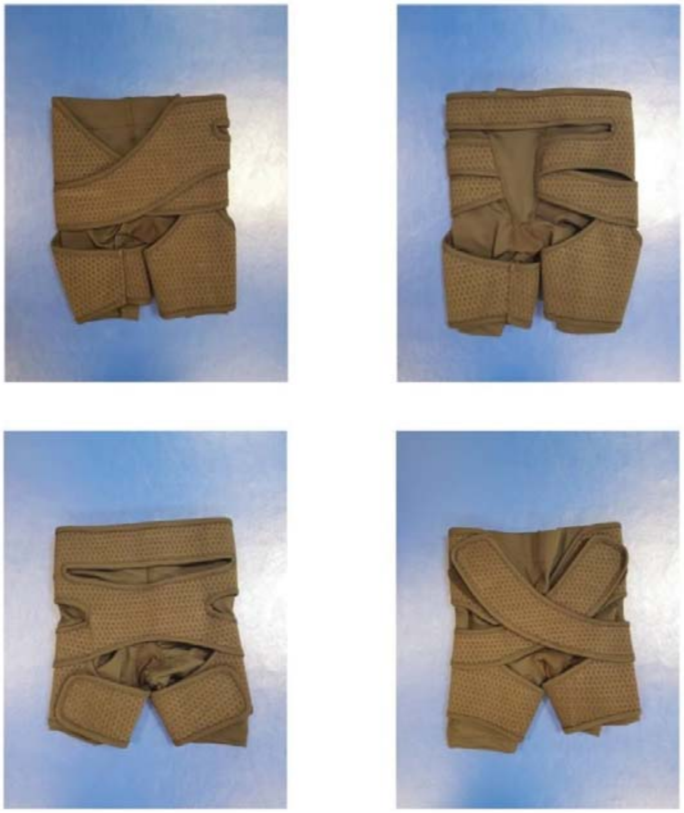

<2nd prototype>

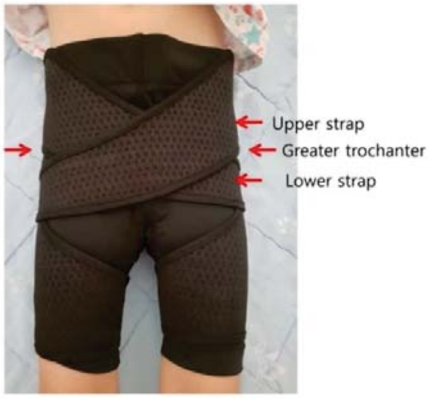

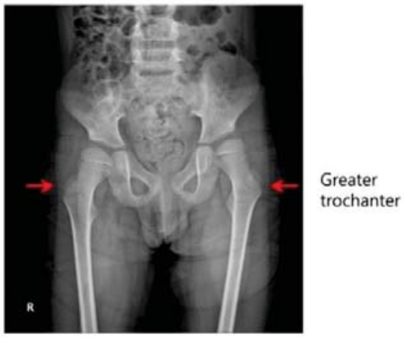

<3rd prototype>

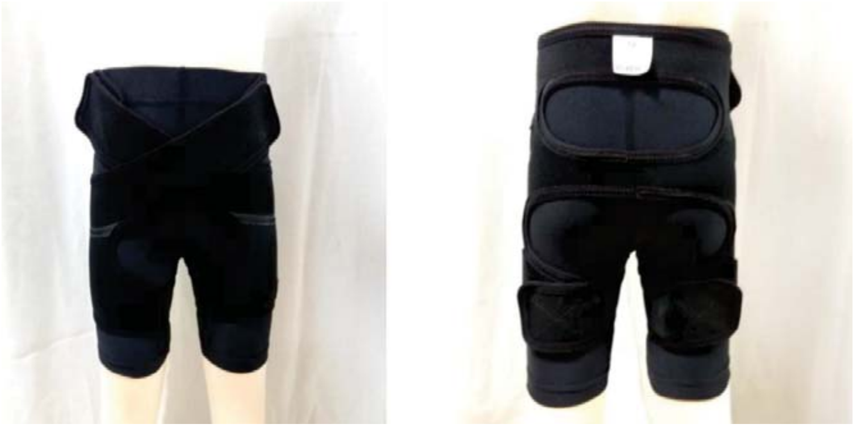

<4th prototype>

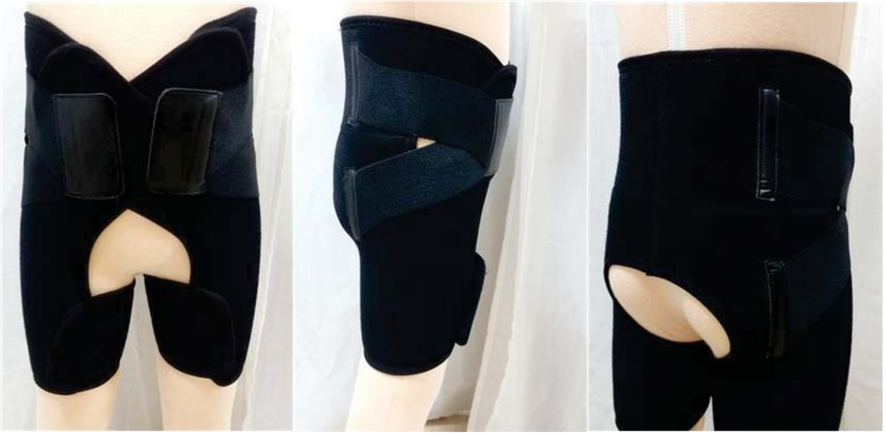

10. Selection criteria, exclusion criteria, target number of subjects and calculation basis

1) Inclusion criteria

- Over 1 year of age and under 10 years of age
- Those with GMFCS level IV or V among children with severe cerebral palsy
- Those who have had quadriplegia or paraplegia for more than 6 months
- hip subluxation /dislocation in children with spastic cerebral palsy occurred before the age of 1 year, so we studied 6 8 children aged 1 to 10 years set.

2) Exclusion criteria

- If you do not consent to the study
- Those who are judged to be difficult to participate in under the judgment of the principal investigator
- If you have had hip joint surgery or plan to have surgery during clinical trial

4) Target number of subjects and calculation basis

- GMFCS IV and V patients were combined before wearing auxiliary equipment, MI progression / year was measured to be 7.83 ± 8.73% (set as a control group) (APMR 2019, IS Kim, JS Ryu, etc.) when data.)
- In this study, the MI of the experimental group was set to 1.566 ± 8.73%, assuming that the MI decreased by 80% when wearable auxiliary medical devices were worn.
- When the number of study subjects calculation program (PASS 15.0) was used with Level of significance = 5%, Power = 80%, and Type of test = two-sided, the total sample size was calculated as 32 patients per group and 64 patients in total, and 5% of Considering the dropout rate, a total of 68 experimental and control groups will be recruited.

11. Study Subject Recruitment Plan

Patients with severe cerebral palsy who are undergoing treatment during the study participation period or children who have decided to participate in the study after reading the recruitment notice.

12. Specific research methods

Patients diagnosed with severe cerebral palsy were included in the study.

Assignment to experimental group and control group if consent to the study

Subject's body measurements were performed to measure the size of the hip joint wearable medical device.

Radiologic and clinical evaluation were performed to evaluate hip dislocation.

The experimental group manufactured and applied hip-joint wearable medical devices, continue existing rehabilitation treatment.

The control group continued the existing rehabilitation treatment.

6 months and 1-year, clinical evaluation and radiological evaluation were performed to determine the effectiveness.

11) Control group setting and randomization method

Experimental group and the control group, a block randomization method is adopted.

Randomization was performed using Microsoft Excel program. A total of 68 study subjects (34 in the experimental group, 34 in the control group) were randomly assigned to a block with a block size of 4 and an allocation ratio of 1:1. In addition, in order to stratify GMFCS IV and V, 68 GMFCS IV and V are assigned respectively.

In the header of cells C4, D4, E4, and F4 of the Excel spreadsheet, enter Sequence, Random number, Block, and Group, respectively. ① Enter the numbers from 1 to 68 in cell C, which is the Sequence column, in order, and ② set the seed (GMFCS IV: 11074, GMFCS V: 11075) using the random number generation function of the data analysis tool for reproducibility in D of the Random number column. to assign a random function. ③ In Block column E, repeat the numbers from 1 to 17 in order. ④ In the Group column, input the experimental group and the control group by 34 consecutively. Then, drag from C4 to F4 to select it, and select Home -> Sort & Filter -> Filter. When a scroll bar appears in each cell, select ascending sort by clicking the scroll bar of E4, and then click the scroll bar of E4 to sort in ascending order to complete the randomization table (Kang, 2017).

12) Multi-center Data Analysis Methods

Total number of study subjects is 68, and Jeju National University Hospital, Pusan National University Hospital, Bundang Jesaeng Hospital, and Korea University Hospital as well as this institute competitively recruit research subjects. The randomization of study subjects is handled by the randomization officer of this institution, and when enrolling research subjects at each institution, randomization numbers are sequentially assigned to determine the experimental group and control group. In addition, the study subjects assigned to the military About The information is random from this institution. Assignment Only the person in charge, the researcher in charge of each institution, the therapist knows, and research subjects, independent Evaluators and Statistical Analysts blindfolded maintain.

To prevent efficacy bias in the analysis, the principal investigator of this clinical trial and the principal investigator of each institution shall not be involved in the series of patient screening and assignment processes. Screening all X-ray images are stored during follow-up and blinding to 3 evaluators who are blindfolded. The analyzed X-ray image is transmitted.

13) Exam drug administration/use, administration/use method, combination therapy, control reason for choosing the drug when using it

None.

14) Observation items, clinical examination items, and observational examination methods

1. Primary efficacy endpoint: hip dislocation through radiographic evaluation (%)

2. Secondary efficacy endpoints: degree of scoliosis through plain radiograph; Degree of femur anteversion, pain index, Functional changes such as satisfaction with wearable medical devices for hip joint protection, hip and knee joint range of motion, discomfort when caring for a patient, and changes in the patient's overall quality of life were investigated.

(4) Hip dislocation test

Hip subluxation and dislocation can be evaluated by plain X-ray (Hip AP radiography). Migration index, which measures the proportion of the femoral head not covered by the hip acetabulum, is widely used. Measured at initial, 6 months, and 12 months.

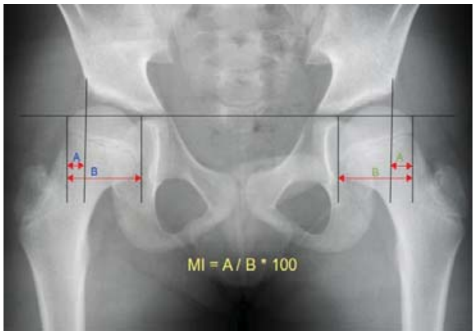

Hip migration index

(2) Degree of scoliosis

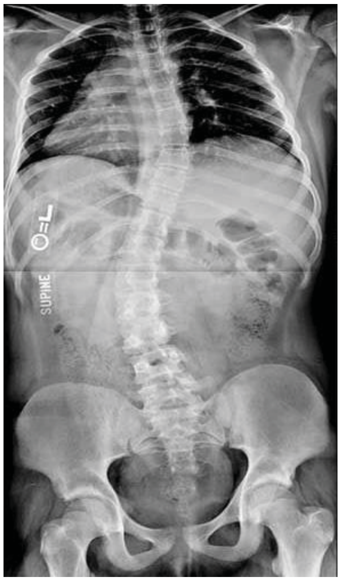

To examine the effect of hip fixation on scoliosis, the whole spine AP is taken while sitting in a sitting chair. The degree of scoliosis is measured by the Cobbs angle. Measured at initial , 6 months, and 12 months .

(3) Femur anteversion

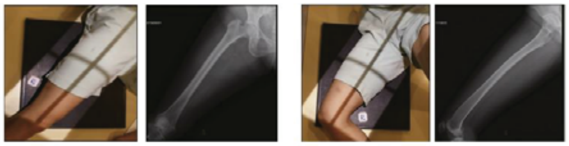

Fig. 1. Anterior-posterior (AP) and lateral (LAT) imaging protocols. In the LAT view, the pelvis is rotated slightly posteriorly to prevent overlap with the femur head.

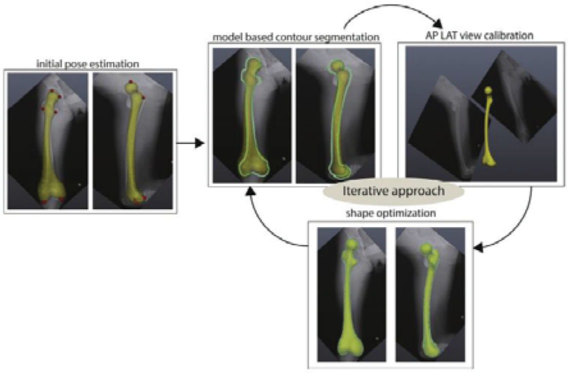

Fig 2. System overview: the anterior-posterior (AP) and lateral (LAT) views are initially pose-estimated using user inputted six points and its 3D correspondences. The image contour is segmented based on the projected contour of the 3D femur. The AP and LAT views are calibrated more accurately using contour correspondences. The shape is optimized so that it projected contour to match the segmented contour. These steps are iteratively performed until convergence.

In the case of Femur anteversion , CT is usually taken, but it has a large amount of radiation exposure. Rather than this , we decided to use a method to measure the anteversion of the femur using simple radiographic examinations such as femur AP and femur lateral x-ray. (K. Youn et al) Measured at the initial stage, 6 months, and 12 months.

(4) Joint range of motion

Using a goniometer Measure the range of motion of the hip and knee of the patient

VI. Hip abduction with hip at 90' flexion

VII. Hip abduction with hip at 0' flexion

VIII. Hip adduction with hip at 0 ' flexion

IX. Hip flexion contracture (Thomas test)

X. Popliteal angle

(5) Questionnaire evaluation

IV. Pain index (Visual analog scale): In the case of children with cerebral palsy GMFCS IV and V communication is difficult, Assess the patient's hip pain felt by the caregiver.

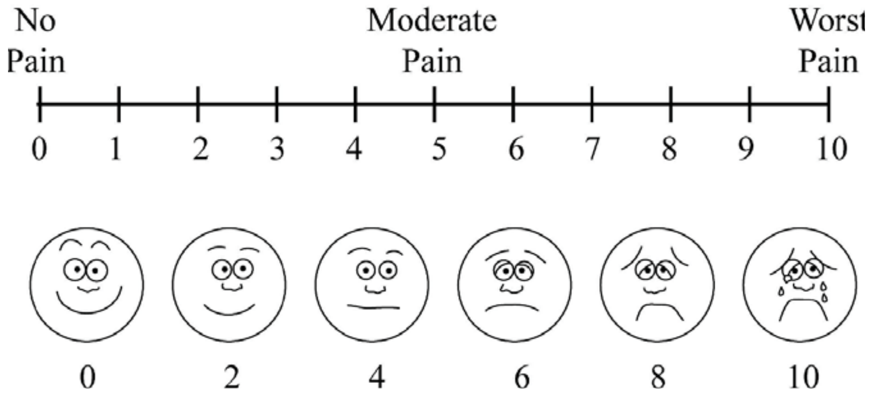

V. Likert scale

A. Evaluating the satisfaction of wearable medical devices for hip joint protection

B. Example of this study questionnaire)

Very satisfied - Satisfied - Moderate - Somewhat dissatisfied - Very dissatisfied

VI. Child Health Index of Life with Disabilities (CPCHILD)

A. As a tool to evaluate the quality of life of patients with severe cerebral palsy It is a tool consisting of 9 items, evaluated by the caregiver. Each item is scored on a scale of 0 to 5, and each item is as follows.

B. daily activities, postures/movements, comfort and emotions, overall, of life quality, the child's of life vaginal Related of the item Investigate items related to hip contracture among CPCHILDs such as importance

C. example

13. From existing treatments and research

To date, there is no established treatment method to prevent hip joint disease.

There are no medical devices to prevent hip dislocation.

14. Benefit and Risk of Study Subjects

(4) Benefit of study subjects

Hip dislocation as an examination in clinical trials to be diagnosed with the current state of the hazard Number and provide detailed training on how to manage it.

(5) Expenses for treatment of study subjects

There is no additional cost to be paid by the study subject, as the Investigator's cost for clinical evaluation, radiological examination, and orthosis is borne by the Investigator.

(6) Forecast side effects/risks and countermeasures

Simple X-ray imaging is an examination method with proven safety. In addition, in the case of children with spastic cerebral palsy, periodic plain X-rays are recommended to evaluate the risk of dislocation (GMFCS IV 4 at 9-month intervals, GMFCS V at 6 month intervals is recommended for surveillance purposes standard of care). Does not increase the risk.

Because the hip dislocation auxiliary medical device is in the form of trousers, special side effects are not predicted, but if a side effect occurs, the treatment cost is paid by the study fund.

15. Criteria for suspension and dropout

(3) Justice

Subjects who have completed the late evaluation according to the clinical trial protocol are considered to have completed the trial. The completion of the trial of all subjects participating in the clinical trial should be recorded, and if the visit or observation was interrupted, the reason should be stated.

(4) Criteria for stopping clinical trials

V. If the circumstances observed during the clinical trial make it unreasonable to proceed with the clinical trial, the principal investigator must request the clinical trial to be suspended to the clinical trial review committee can be stopped.

VI. **Investigator** wishes to suspend a clinical trial for reasons such as safety of a clinical trial medical device, he/she may request to suspend the clinical trial to the clinical trial review committee and suspend the clinical trial according to the decision of the clinical trial review committee.

VII. In case of temporary suspension for the treatment of adverse events that have occurred

VIII. Suspension due to the occurrence of serious adverse events and abnormal medical device reactions

(5) Discontinuation of clinical trials

clinical trial stopped case The clinical trial director stopped up to the point proceeded A11 clinical

trial related data in summary The principal investigator will keep it and discard it according to the procedure.

III. When a clinical trial is suspended, the clinical trial director must organize and deliver the case record, clinical trial progress, and results for the subjects who have progressed up to the time of the suspension to the principal investigator and return all test-related data (case records) to the principal investigator.

IV. If the clinical trial is discontinued, the clinical trial director must immediately notify the subject of the discontinuation of the trial, ensure that appropriate measures and follow-up can be made, and notify the trial review committee in writing.

(4) The subject's leaving out standard

X. If the study is discontinued at the request of the subject or his/her representative

XI. Clinical trial to the result Affect line Number there is surgery, drug or medical device in parallel used case

XII. Clinical trial properly not done not case

XIII. Subject's by absence Inability to conduct early and late evaluations case

XIV. Withdrawal of consent at the request of the subject or his/her representative

XV. In case tracking is not possible due to non-scheduled visit

XVI. In case the subject dies due to reasons not related to the clinical trial

XVII. When the investigator determines that the suspension of the test is beneficial to the subject

XVIII. If the other investigator determines that there is a problem with the clinical trial

On the other hand, if the study is stopped due to safety problems due to the occurrence of adverse events or worsening of complications, the principal investigator shall take appropriate measures and follow -up until possible symptoms are recovered. Relevant information should be recorded in the case record.

(5) Investigator dropout

IV. If a subject is dropped out, the reason for dropping out and the data related to the clinical trial conducted before the dropout shall be recorded and kept.

V. Dropouts are included in statistical processing through safety and efficacy evaluation

unless there is a justifiable reason or justification.

VI. For data obtained through clinical trials, a data set for efficacy analysis and safety analysis should be established.

16. Evaluation criteria for safety including side effects, evaluation methods and reporting methods

(6) Definition of Adverse Events

What is an 'Adverse Event (AE)'? clinical trial middle in the subject occurred every not intended not sign, laboratory Experiment of the result More than My back include), symptoms, or disease say, that clinical trials and must causal You don't have to have a relationship.

(7) Assessment of adverse events

III. severity assessment

More than case of moderate Grades 1-3 in 3 steps into Evaluate.

Grade	Severity	Evaluation standard
Grade 1	Mild	The degree to which the subject does not interfere with normal daily life (function) to a degree that is hardly felt. The extent to which most treatment is not required
Grade 2	Moderate	The degree to which the research subject may feel discomfort, and the degree to which normal daily life (function) is impaired. The degree to which the study subject may continue the trial but may require treatment
Grade 3	Severe	The degree to which the study subjects are very uncomfortable and daily life (function) is impossible, and continuous participation in the test is impossible. The extent to which treatment or hospitalization may be required

IV. Evaluation of causal relationships with medical devices for clinical trials

More than case manifestation city clinical for testing with medical devices correlation Whether doctor in charge next and together Classify and test of the person in charge opinion add up

-Definitely related

The relationship between the occurrence of an adverse event and the use of a medical device for clinical trials is valid, and it is most likely explained by the use of the medical device for clinical trials than any other reason, and disappearance of an adverse event caused by the discontinuation of the medical device for clinical trials, and re-appearance of an adverse event caused by reusing the medical device. In addition, the symptom of adverse event is consistent with information already known about the medical device for the clinical trial or the medical device of the same class.

-Probably related

There is evidence that the relevant clinical trial medical device is used, the time sequence of the use of the clinical trial medical device and the occurrence of an adverse event is reasonable, and it is explained more probably by the use of the medical device for the clinical trial than other causes. When symptoms of abnormality caused by the discontinuation of the use of medical devices for testing disappear

-Relevance is suspected.

There is evidence that a clinical trial medical device has been used, and the time sequence of the use of the clinical trial medical device and the occurrence of an adverse event is reasonable, and is judged to be due to the use of the clinical medical device at the same level as other possible causes. If the symptoms of anomalies caused by the discontinuation of the medical device for clinical trial disappear

-Probably not related

There is evidence that the medical device for the clinical trial is used, there is a more probable cause for the adverse event, and the symptoms of the adverse event caused by the discontinuation of the medical device for the clinical trial disappear or are ambiguous, and the reuse (only when reusable) result of the medical device for the clinical trial is adverse event or symptom is unclear or ambiguous

Definitely not related

-If the clinical trial medical device is not used, or the time sequence between the use

of the clinical trial medical device and the occurrence of the adverse event is not valid or there are other obvious causes for the adverse event.

-Unable to evaluate (Unknown)

When information cannot be judged due to insufficient or conflicting information and cannot be supplemented or verified

(8) Evaluation criteria for safety

by subject in the case record described Adverse events, vital sign, laboratory test result consider evaluation

(9) How to evaluate safety

Vital signs and laboratory tests: For each evaluation, discomfort and skin problems are evaluated at each visit, and a safety evaluation questionnaire is filled out after the test.

(10) How to report adverse events

III. Adverse case

Adverse case of anomalies summary and analysis is for clinical trials Medical Equipment apply after occurred Adverse events (TEAE) about Analyz.

Application group not really Adverse events (AE), medical devices Adverse event (ADE), serious Adverse events (SAE) Descriptive statistics for incidence and manifestation number of cases) present between application groups Adverse events (AE), medical devices Adverse event (ADE), serious Adverse event (SAE) incidence. The difference whether Pearson's chi-square test or by Fisher's exact test analyze.

Expressed every Adverse events (AE), medical devices Adverse events (ADEs) and serious Adverse events (SAE) about MedDRA latest version using System Organ Class (SOC) and Preferred Term (PT) according to to code coded Adverse case number of expression subjects, expression rate and the number of manifestations by county present. Also serious in an abnormal case about details write

IV. Serious adverse events

Serious abnormal case safety follow up term within occurring If , over case of Whether or not related to clinical trials Clinical Investigator or the test officer 24 hours to the Korea Textile Development Institute within report , and term within to the IRB have to report do _

17. Effect evaluation criteria, evaluation method and interpretation method (statistical analysis method, etc.)

All X-ray readings are performed by 3 specialists in a blinded state, and all are analyzed and the average value of the overall evaluation results is used.

(1) Data analysis and statistical analysis method

1. Primary efficacy endpoint: hip dislocation through radiographic evaluation (%)
2. Secondary efficacy endpoints: degree of scoliosis through plain radiograph; pain index, Functional changes such as satisfaction with wearable medical devices for hip joint protection, hip and knee joint range of motion, discomfort when caring for a patient, and changes in the patient's overall quality of life were investigated.

The clinical evaluation is statistically compared between the initial and simple X-ray results after 1 year (compare with paired t-test or Wilcoxon signed rank test according to normality test). In addition, the difference in change values for the initial, 6 months, and 1 year after simple X-ray imaging and clinical evaluation is statistically compared. Repeatedly measured values are verified by a linear mixed model or a generalized estimating equation (GEE) between the experimental group and the control group. If the P-values are < 0.05, it is judged to be a significant level.

Dropout, all data including in statistical processing (ITT) will be analyzed through safety and efficacy evaluation, unless there is a valid reason or justification. When missing values occur in the FAS group, they are excluded and analyzed because it is difficult to satisfy the assumption of Missing Completely at Random (MCAR).

18. Treatment and treatment standards of study subjects after clinical trials

After clinical trial, the subject will follow the further treatment plan of the hospital, the subject should pay the further cost of the treatment. Otherwise, in case of side effects occurred during the trials, and the causality with medical devices is confirmed the principal investigator should

offer the payment of the treatment.

In patients who drop out or had no reaction during the trial, the clinician should guide the subject to receive the appropriate treatment. The doctor should give instructions to re-visit the hospital at any time in case of unexpected delayed adverse effect.

19. Study schedule

IRB approval to 2 years, among children with spastic cerebral palsy, test subjects were recruited, tested, and data were analyzed.

Item	Screening	Before wearing auxiliary medical devices	180 days	360 days
		Visit 1	V isit6	end visit
Visit Window	1 week	-	within ± 4 weeks	± 4 weeks Within
written consent	O	X	X	X
Selection/Exclusion Criteria	O	X	X	X
body measurements	O	X	X	X
medical history	O	X	X	X
Frequency and type of rehabilitation treatment	O	X	X	X
clinical exam: X-ray (Whole spine AP, Total Hip AP , Femur AP/Lat) hip migration index and Cobbs angle , Femur	X	O	O	O

in at io n	anteversion measure)				
	Hip, knee ROM	X	O	O	O
	Satisfaction (Likert scale)	X	X	O	O
	CPCHILD Poll	X	O	O	O
	Adverse reaction inspection	X	O	O	O
	Pain Index (VAS)	X	O	O	O
Medical device manufacturing (size measurement)		O	X	X	X
Start wearing medical devices			O	O	O

If there is an X-ray result within 1 month, the existing data is used for the study.

20. Data and Stability Monitoring Plan (DSMP)

1) monitoring officer

(1) For data and safety monitoring: Jaewon Beom, Principal Investigator

(2) Data and safety monitoring: Person in charge of Seon Cho

2) **Monitoring data and safety information**

Review of key efficacy endpoints for decision to continue, change, or discontinue the study

① No interim analysis of effectiveness

② The safety review is carried out as follows, and the decision to change or discontinue is made.

I. When a serious and unexpected adverse event occurs

(A) Decision to change or discontinue the study after review by the principal investigator

(B) In case of notification of change or suspension of research as a result of the report by the Bioethics Review Committee

II. Safety review once every 6 months

(A) The lead investigator decides to change or discontinue the study after reviewing all adverse reactions that occurred once every 6 months

reported to the Bioethics Review Committee of each institution

III. Safety items: pain and discomfort before and after wearing the orthosis, skin change

③ Data and safety Monitoring method and frequency: quarterly (6 months)

④ Reporting of adverse drug reactions, non-compliance with research, and reporting of unexpected problems

Jae-won Beom is designated and managed as the monitoring manager, and the principle investigator and working person in charge conduct monitoring every 6 months. In case of a serious adverse reaction occurring during the course of the study, report it immediately to the IRB.

(1) Medical device adverse reaction report (fatal/life-threatening): Death or life-threatening cases within 7 days (initial report) [based on working days]

(2) Medical device adverse reaction report (not fatal/life-threatening): other than hospitalization or extension of hospitalization period; resulting in persistent or significant disability or dysfunction; cause congenital anomalies or abnormalities; important medical events, Etc.) Case within 15 days [based on working days]

(3) Report safety-related information: Collect and report at least once every 6 months

(4) Report unexpected problems: within 15 working days

(5) Report material non-compliance: within 15 working days

(6) Reporting of Non-Significant Non-compliance: At least once a year

⑤ Criteria for study discontinuation

(1) If the number of investigators cannot be recruited within the IRB approval period, it is decided whether to suspend or alter the study.

(2) When the study is discontinued, the study subjects follow the treatment procedures of the hospital in relation to treatment.

21. Study subjects safety protection for Measures

1) **Basic measures for securing research ethics**

This study complies with the Declaration of Helsinki (revised in 2013) and ICH-GCP, and the study is conducted after IRB approval.

2) **Consent process of study subjects**

- Principal Investigator who will explain to the subject and obtain consent: Jaewon Beom, Jiwoon Lim , Hyeonjeong Lee, Jihyeon Seo, Boryun Kim, and Yongbeom Shin
- Persons to provide consent: study subjects and representatives
- Time between study description process and consent acquisition process: less than 3 days
- Method to minimize the possibility of coercion or undue influence: In order to minimize the possibility of coercion or undue influence, the decision to participate in the study is voluntary, and you can refuse to participate in the study or freely stop participating at any time during the study period. Therefore, you will not receive any disadvantages in your next treatment, and you may withdraw your consent to participate in the study at any time.
- Language used by principal investigator in the process of explaining the study and obtaining consent: Korean
- Language that the study subject or representative can understand: Korean
- Information provided to research subjects or their representatives and consent form: Provide consent form and explanation

3) **Compensation plan for study subjects**

- Transportation expenses will be provided when visiting the hospital for study, and it will be completed with an average of 3 visits. At the end of the study, transportation expenses are added up and 120,000 won is paid to the patient's guardian's account. In the case of the control group, two sets of auxiliary medical devices are provided when the experiment is finished.

4) Measures to protect personal information of study subjects

The personal information of study subjects is protected and managed through initials and subject selection code. Through this, it is managed so that personal identification is impossible. Alternatively, study data is stored in a password-protected file and stored in a locked laboratory. In accordance with Article 15 of the Enforcement Regulations of the Bioethics Act, study-related records must be kept for 3 years from the time the study is completed, and documents past the storage institution must be destroyed in accordance with Article 16 of the Enforcement Decree of the Personal Information Protection Act, and related data are If storage is required for more than 3 years for accumulation, etc., report it to the IRB and keep it.

5) Reasons for including vulnerable study subjects (children)

In patients with cerebral palsy, hip dislocation mainly occurs in children, and hip dislocation occurs around the age of 10 years of age after hip dislocation surgery.

6) Additional protection measures in case of inclusion of vulnerable study subjects

Considering that patients with cerebral palsy do not have adequate cognitive ability to consent to the study due to cognitive decline, In the case of study subjects, pediatric consent is exempted, and the study will be conducted with the consent of one of the parents and parents of all study subjects.

*GMFCS level IV and V patients participating in this study are quadriplegia patients and cannot speak without following the 1st step instructions.

22. How to store and dispose of human materials

None

23. Note literature

1. Jeongrim Moon, Im Ji-eun, Young- Wan Moon , Song Dae-heon. Frequency of hip dislocation according to type and motion index in children with cerebral palsy . Journal of Korean Rehabilitation Medicine 2004.

2. Park Joo-hyun; Kang Se- yoon , Yoon Yeon- joong , immature, A study on hip joint

deformity in children with cerebral palsy . Journal of Korean Rehabilitation Medicine 1996.
3. Miller SD, Juricic M, Hesketh K, et al. Prevention of hip displacement in children with cerebral palsy: a systematic review. Dev Med Child Neurol. 2017.
4. Parrot J, Boyd RN, Dip PG, Dobson F: Hip displacement in spastic cerebral palsy: repeatability of radiologic measurement. J Ped Orthop 2002.
5. K. Youn et al., Iterative approach for 3D reconstruction of the femur from un-calibrated 2D radiographic images, Medical Engineering and Physics (2017)

Study plan change comparison table

Study project name: The Clinical Efficacy of hip protection orthosis for the protection of hip dislocation in patients with severe cerebral palsy, single blinded, randomized-control trial, multicenter clinical trial

v2.0

Item	Details / page	Before change	After change	Note
Study plan	Title	(국문) 중증 뇌성마비 환자의 고관절 탈구 방지를 위해 개발된 고관절 보호 착용형 의료기기의 임상적 유효성 검증을 위한 다기관 임상시험<br>(English) The Clinical Efficacy of Hip Protection Orthosis for The Protection of Hip Dislocation in Patients with Severe Cerebral Palsy, Single Blinded, Open Label, Multicenter Clinical Trial	(국문) 중증 뇌성마비 환자의 고관절 탈구 방지를 위해 개발된 보호 착용형 의료기기의 임상적 유효성 검증을 위한 무작위 배정, 다기관 전향적 임상시험<br>(English) The clinical efficacy of hip protection orthosis for the protection of hip dislocation in patients with severe cerebral palsy, single blinded, randomized-control trial, multicenter clinical trial	
	Background and purpose of the study<br>(2) Problem of hip dislocation in children with cerebral palsy<br>p.5	stylus variant	spiky deformity	typo correction
	Target number of subjects and calculation basis<br>p.8-9	- this study, assuming that the MI decreased by 40% for GMFCS IV and 50% for GMFCS V when wearable auxiliary medical devices were worn, the MI of the experimental group was set to 15.58±9.28 and 13.45±8.86, respectively.<br>- With Level of significance = 5%, Power = 80%, Type of test = two -sided research subject Number Calculation When using the program (G-power 3.1.9.2), the total sample size is that of GMFCS IV . case experimental group control 16 each, GMFCS V _ experimental group control with 12 people each calculated, a total of 56 experimental group control group to recruit planned.	- this study, assuming that the MI decreased by 40% for GMFCS IV and 50% for GMFCS V when wearable auxiliary medical devices were worn, the MI of the experimental group was set to 16.134±17.1 and 13.445±17.1, respectively.<br>With Level of significance = 5%, Power = 80%, Type of test = two -sided research subject Number Calculation When using the program (G-power 3.1.9.2), the total sample size is that of GMFCS IV. case experimental group control 11 each, GMFCS V _ experimental group control 8 people each was calculated, and a dropout rate of 10% was Taking this into account, a total of 42 experimental group control group to recruit planned.	As recommended, the number of subjects was recalculated by correcting the mean and standard deviation values.
	Control group setting and randomization method<br>p.9	The experimental group was the patients receiving treatment at Seoul National University Bundang Hospital.<br>Control group setting: Subjects were treated at Jeju National	To prevent an imbalance in the number of study subjects between the experimental group and the control group, a block randomization method is adopted.	Changed the study design to randomization and added a description of the randomization

		University Hospital.<br>Randomization Method: Not applicable.	Randomization was performed using Microsoft Excel program. 24 GMFCS IV subjects and 18 GMFCS V subjects were randomly assigned to two groups with a block size of 4 and an allocation ratio of 1:1. In the header of cells A1, B1, C1, D1 in the Excel spreadsheet, enter Sequence, Random number, Block, Group. each Enter. ① Enter the numbers 1 to 24 and 1 to 18 in cell A , the sequence column . In order Enter, ② in the Random number column in B function = Rand ( ) by typing random function allocate _ ③ In Block column C, repeat the numbers 1 to 6 and 1 to 4 in order. At this time, the numbers 1 to 4 or 6 are repeated 4 times, which means that 24 and 18 study subjects were assigned a block size of 4. ④ In the Group column, enter the experimental group and control group, respectively, followed by 12 and 9 people. Then, drag from B1 to D1 to select it, and select Home -> Sort & Filter -> Filter. When a scroll bar appears in each cell, click the scroll bar of C 1 to select ascending sort, and then click the scroll bar of C 1 to sort in ascending order to complete the randomization table (Kang, 2017). A random number table is created, and a new random function is generated at the start of the study. At this time, the random number on the sheet may change and the assignment may be changed, so copy or capture the value and keep it.<br>**Multicenter Data Analysis Methods**<br>total number of study subjects is 42, and not only this institution but also Jeju National University Hospital recruits research subjects competitively. The randomization of study subjects is handled by the randomization officer of this institution, and when enrolling research subjects at each institution, randomization numbers are sequentially assigned to determine the experimental group and control group.	method.
	questionnaire evaluation<br>P.11 _	As a tool to evaluate the quality of life of patients with severe cerebral palsy It is a tool consisting of 9 items. Each item is scored on a scale of 0 to 5, and each item is as follows.	As a tool to evaluate the quality of life of patients with severe cerebral palsy It is a tool consisting of 9 items. evaluated by the caregiver, each item is scored on a scale of 0 to 5, and each item is as follows.	An explanation has been added to the questionnaire evaluated by the guardian.

	reports of adverse drug reactions, non- compliance with research , Reporting unexpected problems<br>p.19	(7) serious non-compliance Within 15 days of occurrence<br>(8) Minor plan non-compliance Occurrence: Once a year<br>(9) abnormal drug reaction Within 15 days of occurrence<br>(10) not expected unhappy Problem Within 15 days of occurrence<br>(11) safety relation information Report: Once a year	(1) Medical device adverse reaction report (fatal/life-threatening): Death or life-threatening cases within 7 days (initial report) [based on working days]<br>(2) Medical device adverse reaction report (not fatal/life-threatening): other than hospitalization or extension of hospitalization period; resulting in persistent or significant disability or impairment; cause congenital anomalies or abnormalities; important medical events, Etc) Case within 15 days [based on working days]<br>(3) Report safety-related information: Collect and report at least once every 6 months<br>(4) Report unexpected problems: within 15 working days<br>(5) material non- compliance: within 15 working days<br>(6) Non-Significant Non- Compliance Cases: More than once per year	Modified as recommended.
	Additional protection measures in case of inclusion of vulnerable research subjects<br>p. 21	of understandable difficulty Create a consent form to ' provide and understand information' help. In addition, to guarantee the consent process and spontaneity, it is planned to reflect children's wishes as much as possible, and to proceed with oral consent or consent waiver for children under the age of 7 years. For those over 7 years of age, obtain the attached pediatric consent form. The consent of one of the parents was obtained from all study subjects. Considering that patients with cerebral palsy do not have adequate cognitive ability to consent to the study due to decreased cognitive ability, participating pediatric ability Because it is very limited, About Infant of the study subject comment reasonably refer to Number Do not have In this case, the child's consent is waived.	Considering that patients with cerebral palsy do not have adequate cognitive ability to consent to the study due to cognitive decline, In the case of research subjects, pediatric consent is exempted, and the research will be conducted with the consent of one of the parents and parents of all research subjects.	According to the selection criteria of subjects to participate in this study, voluntary consent is difficult due to reduced cognitive ability. We plan to proceed with the consent of one of the parents.

V3.0

Item	Details / page	Before change	after	note
research plan	Additional protection measures in case of inclusion of vulnerable research subjects	Considering that cerebral palsy patients do not have adequate cognitive ability to consent to the study due to cognitive decline, the research subject will be exempted from pediatric consent, and the study will be conducted with the consent of one parent or parent of all study subjects.	Considering that cerebral palsy patients do not have adequate cognitive ability to consent to the study due to cognitive decline, the research subject will be exempted from pediatric consent, and the study will be conducted with the consent of one parent or parent of all study subjects.<br>*GMFCS level 4,5 patients participating in this study are quadriplegia patients and cannot speak without following the first step instructions.	Impaired consent ability is expected to use the 1st stage of following instructions, and in the case of this study subject, 1st stage of following instructions is not available.

v4.0

<table>
<tr><th>Item</th><th>Details / page</th><th>Before change</th><th>after</th><th>note</th></tr>
<tr><td></td><td>Major Selection Exclusion Criteria
p.3, 8</td><td>botulinum injections up to 3 months ago or are planning to undergo treatment during clinical research</td><td>delete</td><td>Willoughby et al. According to a 2012 paper, botulinum injection was found to be ineffective, so it was deleted from the exclusion criteria.</td></tr>
<tr><td></td><td>Joint research institutes and principal researchers of the institutes
p.4</td><td><table><tr><th>statement</th><th>Material Organization name</th><th>Major</th><th>spot</th><th>contact</th></tr><tr><td>Bo Ryun Kim</td><td>Jeju National University Hospital</td><td>Department of Rehabilitation Medicine</td><td>associate professor</td><td>010-9828-0610</td></tr></table></td><td><table><tr><th>Statement</th><th>Material Organization name</th><th>Major</th><th>spot</th><th>contact</th></tr><tr><td>Bo Ryun Kim</td><td>Jeju National University Hospital</td><td>Department of Rehabilitation Medicine</td><td>associate professor</td><td>010-9828-0610</td></tr><tr><td>Jee Hyun Suh</td><td>Bundang Jesaeng Hospital</td><td>Department of Rehabilitation Medicine</td><td>Professor</td><td>010-6528-6578</td></tr></table></td><td>A joint research institute has been added.</td></tr>
<tr><td></td><td>Target number of subjects and calculation basis
p.8-9</td><td>- existing copy researcher's in research assistant equipment to wear The MI of all GMFCS IV and V patients was 26.89±17.10%. Measured (as control Settings) (APMR 2019, IS Kim, JS Ryu , etc.)
- copy in research wearable assistant medical device would have worn When MI decreases by 40% for GMFCS IV and 50% for GMFCS V assuming MI of the experimental group 16.134±17.1 and 13.445±17.1, respectively.
- With Level of significance = 5%, Power = 80%, Type of test = two -sided research Calculate the number of subjects When using the program (G-power 3.1.9.2), the total sample size is that of GMFCS IV. case experimental group control 11 each, GMFCS V experimental group control It was calculated as 8 people each, and the dropout rate was 10% . Taking this into account, a total of 42 experimental groups and control group to recruit planned.</td><td>- In the previous study of this researcher, the MI of GMFCS IV and V patients before wearing auxiliary equipment was measured to be 26.89±17.10% (set as a control group) (APMR 2019, IS Kim, JS Ryu, etc.)
- In this study, the MI of the experimental group was set to 13.445±17.1 and 16.134±17.1, respectively, assuming that the MI decreased by 50% for GMFCS IV and 40% for GMFCS V when wearable auxiliary medical devices were worn.
- With Level of significance = 5%, Power = 80%, Type of test = two -sided research Calculate the number of subjects When using the program (G-power 3.1.9.2), the total sample size is that of GMFCS IV. case experimental group control 8 people each, GMFCS V experimental group control It was calculated as 11 people each, and the dropout rate was 10%. Taking this into account, a total of 42 experimental groups and control group to recruit planned.</td><td>Previously, it was expected that patients with GMFCS V would see more changes when they wore braces, but when reviewing the literature, with GMFCS V, hip dislocation progressed steeper, so it was judged that the effect of the braces would be less, so the subject calculation was limited. I did it again. The total number of subjects remains the same.</td></tr>
</table>

v5.0

Item	Details / page	Before change	after	note
Calculation of the number of study subjects	Target number of subjects and calculation basis p. 8-9	- In the previous study of this researcher, the MI of GMFCS IV and V patients before wearing auxiliary equipment was measured to be 26.89±17.10% (set as a control group) (APMR 2019, IS Kim, JS Ryu, etc.)<br>- In this study, the MI of the experimental group was set to 13.445±17.1 and 16.134±17.1, respectively, assuming that the MI decreased by 50% for GMFCS IV and 40% for GMFCS V when wearable auxiliary medical devices were worn.<br>- If the number of study subjects calculation program (G-power 3.1.9.2) is used with Level of significance = 5%, Power = 80%, Type of test = two-sided, the total sample size is 8 each for the experimental group and control group for GMFCS IV. The number of persons and GMFCS V was calculated as 11 persons in the experimental group and the control group, respectively . Considering the dropout rate of 10% , a total of 42 experimental and control groups will be recruited.	- In the previous study of this researcher, when GMFCS IV and V patients were combined before wearing auxiliary equipment, MI progression / year was measured to be 7.83 ± 8.73% (set as a control group) (APMR 2019, IS Kim, JS Ryu, etc.) when data.)<br>- In this study, the MI of the experimental group was set to 1.566 ± 8.73%, assuming that the MI decreased by 80% when wearable auxiliary medical devices were worn.<br>- When the number of study subjects calculation program (PASS 15.0) was used with Level of significance = 5%, Power = 80%, and Type of test = two-sided, the total sample size was calculated as 32 patients per group and 64 patients in total, and 5% of Considering the dropout rate, a total of 68 experimental and control groups will be recruited.	As the number of study subjects was calculated incorrectly, we sought advice from the MRCC of our hospital and recalculated it.
Control group setting and randomization method	Control group setting and randomization method p. 9-10	To prevent an imbalance in the number of study subjects between the experimental group and the control group, a block randomization method is adopted.<br>Randomization was performed using Microsoft Excel program. 24 GMFCS IV subjects and 18 GMFCS V subjects were randomly assigned to two groups with a block size of 4 and an allocation ratio of 1:1. In the header of cells A1, B1, C1, and D1 in the Excel spreadsheet, enter Sequence, Random number, Block, and Group, respectively. ① Enter the numbers 1 to 24 and 1 to 18 in cell A, which is the Sequence column, in order, and ② Enter the function = Rand() in B of the Random number column to assign a random function. ③ In block column C, repeat the numbers 1 to 6 and 1 to 4 in order. At this time, the numbers 1 to 4 or 6 are repeated 4 times, which means that 24 and 18 study subjects were assigned a block size of 4. ④ In the Group column,	To prevent an imbalance in the number of study subjects between the experimental group and the control group, a block randomization method is adopted.<br>Randomization was performed using Microsoft Excel program. A total of 68 study subjects (34 in the experimental group , 34 in the control group) were randomly assigned a block size of 4 and an allocation ratio of 1:1. In addition, in order to stratify GMFCS IV and V, 68 GMFCS IV and V are assigned respectively.<br>In the header of cells C4, D4, E4, and F4 of the Excel spreadsheet, enter Sequence, Random number, Block, and Group, respectively. ① Enter the numbers from 1 to 68 in cell C, which is the Sequence column, in order, and ② set the seed (GMFCS IV: 11074, GMFCS V: 11075) using the random number generation function of the data analysis tool for reproducibility in D of the Random number column. to assign a random function. ③ In Block column E, repeat the	

		input the experimental group and the control group, respectively, followed by 12 and 9 people. Then, drag from B1 to D1 to select it, and select Home -> Sort & Filter -> Filter. When there is a scroll bar in each cell, click the scroll bar of C1 to select ascending sort, and then click the scroll bar of C1 to sort in ascending order to complete the randomization table (Kang, 2017). A random number table is created, and a new random function is generated at the start of the study. At this time, the random number on the sheet may change and the assignment may be changed, so copy or capture the value and keep it.	numbers from 1 to 17 in order. ④ In the Group column, input the experimental group and the control group by 34 consecutively. Then, drag from C4 to F4 to select it, and select Home -> Sort & Filter -> Filter. When there is a scroll bar in each cell, click the scroll bar in E4 to select ascending sort, and then click the scroll bar in E4 to sort in ascending order to complete the randomization table (Kang, 2017).	
Research manager and researcher	P. 4.	D. Research officer: Shin Ah-reum<br>E. Researcher: Ah-reum Shin , Seungeun Lee, Eun Gyeong Jang<br>F. Medical device manager for clinical trials: Usually, the in-hospital medical device manager is in charge of medical devices .	D. Research Officer: Seungeun Lee<br>E. Researchers: Lee Seung-eun, Eun Gyeong Jang<br>F. Medical device manager for clinical trials: Usually, the in-hospital medical device manager is in charge of medical devices.	Shin Ah-reum, Lee Seungeun has been changed to teacher.

v 6.0 _

Item	Details / page	Before change	after	note
Research institutes and collaborators	p.4	(-)	2. Name and address of the research institute<br>Department of Rehabilitation Medicine, Pusan National University Hospital: Pusan National University Hospital, 179 Gudeok -ro, Seo-gu, Busan<br>3. Names and titles of the principal investigator and co-researcher<br>C. Joint research institute and the researcher in charge of the institute<br>Shin Bum Shin / Pusan National University Hospital / Department of Rehabilitation Medicine / Professor / 051-240-7485	Professor Yong Beom Shin of Pusan National University Hospital added as a co-researcher
Multi-institutional data analysis method	p.10	Multicenter Data Analysis Methods<br>The total number of study subjects is 68, and not only this institution but also Jeju National University Hospital recruits research subjects competitively.	Multicenter Data Analysis Methods<br>The total number of study subjects is 68, and Jeju National University Hospital and Pusan National University Hospital as well as this institution competitively recruit research subjects.	Professor Yong Beom Shin of Pusan National University Hospital added as a co-researcher
Research schedule	p.18	(-)	Addition of Rehabilitation Treatment Frequency and Type	Add survey content
monitoring officer	p.19	(2) Person in charge of data and safety monitoring: Ah-reum Shin, research manager	(2) Data and safety monitoring manager: Lee Seungeun, research manager	Changed to teacher Seungeun Lee due to the resignation of Ah-reum Shin
Measures for the safety protection of research subjects	Subject's consent process p.20	• Researchers who will explain to the research subjects and obtain their consent: Ju Seok Ryu, Joonyoung Jang, Seungeun Lee, Ah-reum Shin	• Researchers who will explain to the research subjects and obtain their consent: Ju Seok Ryu, Joonyoung Jang, Seungeun Lee	Changed to teacher Seung-eun Lee due to the resignation of Ah-reum Shin
Measures to protect the safety of research subjects	Compensation plan for research subjects p.20	Transportation expenses will be provided when visiting the hospital for research, and it will be completed with an average of 3 visits. At the end of the study, transportation expenses are added up and 60,000 won is paid to the patient's guardian's account. In the case of the control group, one auxiliary medical device is provided when the experiment is finished.	Transportation expenses will be provided when visiting the hospital for research, and it will be completed with an average of 3 visits. At the end of the study, transportation expenses are added up and 120,000 won is paid to the patient's guardian's account. In the case of the control group, two sets of auxiliary medical devices are provided when the experiment is finished.	when changing the “Explanation and consent form for legal representatives” was not reflected in the research plan, so it is amended this time.

v 7.0 _

Item	Details / page	Before change	after	note
Observation items, clinical examination items, and observational examination methods	p.12	(-)	Measured at initial, 6 months, and 12 months.	Added timing for x-ray imaging in relation to Femur anteversion

v 8.0 _

Item	Details / page	Before change	after	note
Specific research methods	p.9	(-)	Medical Equipment the size Right not the subject as a control Assignment .	

v 9.0 _

<table>
<tr><th>Item</th><th>Details
/ page</th><th>Before change</th><th>after</th><th>note</th></tr>
<tr><td>research plan</td><td>p.1</td><td>Principle Researcher Name : Ju Seok Ryu</td><td>Principle researcher Name: Jaewon Beom</td><td>Change of lead researcher</td></tr>
<tr><td>Research outline</td><td>Lead Researcher/p.2</td><td>Ryu, Associate Professor , Department of Rehabilitation Medicine</td><td>Department of Rehabilitation Medicine Jaewon Beom associate professor</td><td>Change of lead researcher</td></tr>
<tr><td>Research outline</td><td>Research method/p.2</td><td>(-)</td><td>To measure the size of the hip joint wearable medical device, the subject's body measurements were performed.</td><td>This item is for measuring the size of the subject's pants orthosis.</td></tr>
<tr><td rowspan="3">research plan</td><td>Principal Investigator and collaborator statement and Title /p.4</td><td>Principal Investigator<table><tr><th>statement</th><th>Location Organization name</th><th>Major</th><th>spot</th><th>contact</th></tr><tr><td>Ju Seok Ryu</td><td>Seoul National University Bundang Hospital</td><td>Department of Rehabilitation Medicine</td><td>associate professor</td><td>031-787-7733</td></tr></table></td><td>Principal Investigator<table><tr><th>statement</th><th>Location Organization name</th><th>Major</th><th>spot</th><th>contact</th></tr><tr><td>Jaewon Beom</td><td>Seoul National University Bundang Hospital</td><td>Department of Rehabilitation Medicine</td><td>associate professor</td><td>031-787-7739</td></tr></table></td><td>Change of lead researcher</td></tr>
<tr><td>Principal Investigator and collaborator statement and Title /p.4</td><td>Researcher: Eun Gyeong Jang</td><td>Researcher: Seon Cho</td><td>Resigned from researcher Eun Gyeong Jang, Joined the Seon Cho</td></tr>
<tr><td>Selection criteria, exclusion criteria, target number of subjects and calculation basis/p.8</td><td>60 children aged 1 to 10 years were set</td><td>68 children aged 1 to 10 years were established</td><td>typo correction (60 -> 68)</td></tr>
</table>

	Specific research methods / p.9	(-)	To measure the size of the hip joint wearable medical device, the subject's body measurements were performed.	This item is for measuring the size of the subject's pants orthosis.
	Data and Stability Monitoring Plan (DSMP)/p.20	data and safety monitoring Responsible for: Ju Seok Ryu lead researcher	data and safety monitoring Responsible person: Jaewon Beom lead researcher	Change of lead researcher
	Data and Stability Monitoring Plan (DSMP)/p.20	Professor Ju Seok Ryu is appointed as the monitoring manager and managed, and the researcher and the person in charge in charge conduct monitoring every 6 months.	Jaewon Beom is designated as the monitoring manager and managed, and the responsible researcher and working person in charge conduct monitoring every 6 months.	Change of lead researcher
	Measures for the safety protection of research subjects/p.21	The researcher who will explain to the research subject and obtain consent: Ju Seok Ryu, Joonyoung Jang, Seungeun Lee	The researcher who will explain to the research subject and obtain consent: JaeWon Beom, Joon -Young Jang	Change of lead researcher

v 10.0

<table>
<tr><th>Item</th><th>Details
/ page</th><th>Before change</th><th>after</th><th>note</th></tr>
<tr><td>research plan</td><td>Name and address of the research institution/p.4</td><td>Department of Rehabilitation Medicine, Seoul National University Bundang Hospital: 82, Gumi-ro 173beon-gil, Bundang-gu, Seongnam-si, Gyeonggi-do, 463-707
Jeju National University Hospital: 15, Aran 13-gil, Jeju-si (064-717-1114)
Department of Rehabilitation Medicine, Pusan National University Hospital: Pusan National University Hospital, 179 Gudeok -ro, Seo-gu, Busan</td><td>Department of Rehabilitation Medicine, Seoul National University Bundang Hospital: 82, Gumi-ro 173beon-gil, Bundang-gu, Seongnam-si, Gyeonggi-do, 463-707
Jeju National University Hospital: 15, Aran 13-gil, Jeju-si (064-717-1114)
Department of Rehabilitation Medicine, Pusan National University Hospital: Pusan National University Hospital, 179 Gudeok -ro, Seo-gu, Busan
Bundang Jesaeng Hospital : 20, Seohyeon-ro 180beon-gil, Bundang-gu, Seongnam-si, Gyeonggi-do
Korea University Hospital: Korea University Hospital, 73 Korea-daero, Seongbuk-gu, Seoul</td><td>Bo Ryun Kim, the lead researcher at Jeju National University Hospital, a joint research institution, moved to Korea University, and the lead researcher of Jeju National University Hospital was changed, and the joint research institution Korea University Hospital was added/ the address of the joint research institution Bundang Jesaeng Hospital was omitted.</td></tr>
<tr><td></td><td>Joint research institutes and principal investigators of the institute/p.4</td><td>
<table>
<tr><th>statement</th><th>Material Organization name</th><th>Major</th><th>spot</th><th>contact</th></tr>
<tr><td>Bo Ryun Kim</td><td>Jeju National University Hospital</td><td>Department of Rehabilitation Medicine</td><td>associate professor</td><td>010-9828-0610</td></tr>
<tr><td>Jee Hyun Suh</td><td>Bundang Jesaeng Hospital</td><td>Department of Rehabilitation Medicine</td><td>Professor</td><td>010-6528-6578</td></tr>
<tr><td>Yong Beom Shin</td><td>Pusan National University Hospital</td><td>Department of Rehabilitation Medicine</td><td>Professor</td><td>051-240-7485</td></tr>
</table></td><td>
<table>
<tr><th>statement</th><th>Material Organization name</th><th>Major</th><th>spot</th><th>contact</th></tr>
<tr><td>Soyoung Lee</td><td>Jeju National University Hospital</td><td>Department of Rehabilitation Medicine</td><td>associate professor</td><td>010-9328-1959</td></tr>
<tr><td>Jee Hyun Suh</td><td>Bundang Jesaeng Hospital</td><td>Department of Rehabilitation Medicine</td><td>Professor</td><td>010-6528-6578</td></tr>
<tr><td>Yong Beom Shin</td><td>Pusan National University Hospital</td><td>Department of Rehabilitation Medicine</td><td>Professor</td><td>051-240-7485</td></tr>
<tr><td>Bo Ryun</td><td>Korea</td><td>Department</td><td>associate</td><td>010-</td></tr>
</table></td><td>Bo Rryun Kim, a researcher at Jeju National University Hospital, a joint research institute, moved to Korea University, and the chief researcher of Jeju National University Hospital was changed and a joint research institute, Korea University Hospital, was added.</td></tr>
</table>

			Kim | University Hospital | of Rehabilitation Medicine | professor | 9828-0610	
	**Multicenter Data Analysis Methods/p.10**	The total number of study subjects is 68, and Jeju National University Hospital and Pusan National University Hospital as well as this institution competitively recruit research subjects. The randomization of study subjects is handled by the randomization officer of this institution, and when enrolling research subjects at each institution, randomization numbers are sequentially assigned to determine the experimental group and control group.	A total of 68 subjects were registered for the study, and not only this institution but also Jeju National University Hospital, Pusan National University Hospital, Bundang Jesaeng Hospital , and Korea University Hospital competitively recruit research subjects. The randomization of study subjects is handled by the randomization officer of this institution, and when enrolling research subjects at each institution, randomization numbers are sequentially assigned to determine the experimental group and control group.	Addition of joint research institute Korea University Hospital, Bundang Jesaeng Hospital added
	**Consent process of study subjects /p.21**	• The researcher who will explain to the research subject and obtain consent: Jae-Won Beom, Joon -Young Jang	• The researcher who will explain to the research subject and obtain consent : Jaewon Beom, Jang Jun-young , So-young Lee, Jee Hyun Suh, Bo Ryun Kim, Yong Beom Shin	Addition of lead researcher of joint research institute

v 11.0

Item	Details / page	Before change	after
research plan	9. Medical devices for clinical trials /p.7	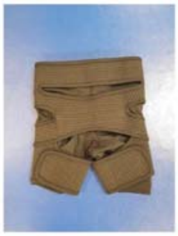 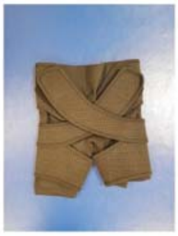 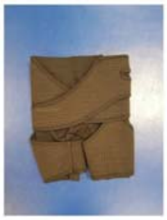 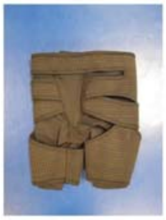 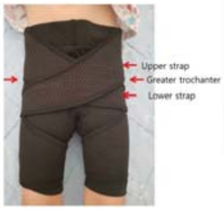 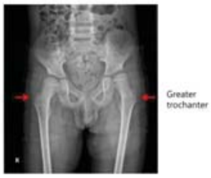	< 1st Prototype ><br>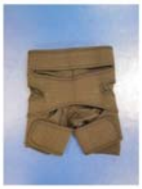 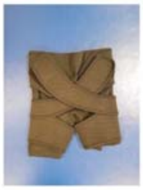 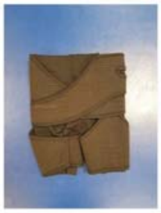 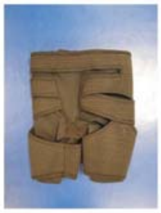<br>< 2nd Prototype ><br>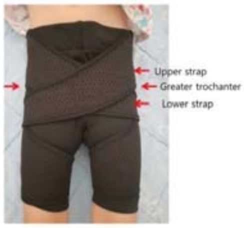 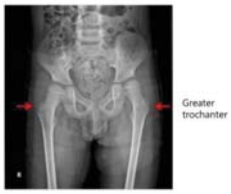 < 3rd Prototype >

			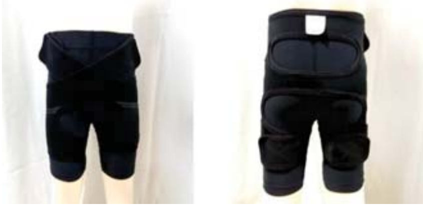<br>< 4th Prototype ><br>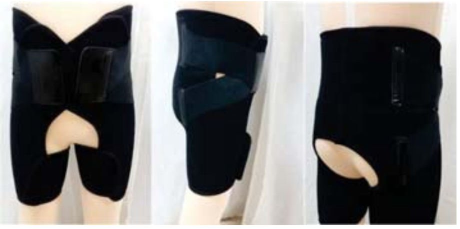

v 12.0

<table>
<tr><th>Item</th><th>Details / page</th><th>Before change</th><th>after</th><th>note</th></tr>
<tr><td>research plan</td><td>Name and title of research director and co-researcher/4p</td><td>Joint research institutes and lead researchers of the institutes
<table><tr><th>statement</th><th>Material Organization name</th><th>Major</th><th>spot</th></tr><tr><td>Soyoung Lee</td><td>Jeju National University Hospital</td><td>Department of Rehabilitation Medicine</td><td>associate profes</td></tr><tr><td>Jee Hyun Suh</td><td>Bundang Jesaeng Hospital</td><td>Department of Rehabilitation Medicine</td><td>Professor</td></tr><tr><td>Yong Beom Shin</td><td>Pusan National University Hospital</td><td>Department of Rehabilitation Medicine</td><td>Professor</td></tr><tr><td>Bo Ryun Kim</td><td>Korea University Hospital</td><td>Department of Rehabilitation Medicine</td><td>associate profes</td></tr></table></td><td>Joint research institutes and lead researchers of the institutes
<table><tr><th>statement</th><th>Material Organization name</th><th>Major</th><th>spot</th></tr><tr><td>Hyun Jeong Lee</td><td>Jeju National University Hospital</td><td>Department of Rehabilitation Medicine</td><td>medical profess</td></tr><tr><td>Jee hyun Suh</td><td>Bundang Jesaeng Hospital</td><td>Department of Rehabilitation Medicine</td><td>Professor</td></tr><tr><td>Yong Beom Shin</td><td>Pusan National University Hospital</td><td>Department of Rehabilitation Medicine</td><td>Professor</td></tr><tr><td>Bo Ryun Kim</td><td>Korea University Hospital</td><td>Department of Rehabilitation Medicine</td><td>associate profes</td></tr></table></td><td>Changed to Lee So-young, co-researcher -> Lee Hyeon-jeong, co-researcher</td></tr>
<tr><td></td><td>Measures for the safety protection of research subjects/22p</td><td>• to the study subjects explain consent to acquire Researcher : Jaewon Beom, Joonyoung Jang, Soyoung Lee, Jee Hyun Suh, Bo Ryun Kim, Yong Beom Shin</td><td>• to the study subjects explain consent to acquire Researcher : Jae -Won Beom , Joonyoung Jang , Hyeon-Jeong Lee , Jee Hyun Suh, Bo Ryun Kim , Yong Beom Shin</td><td>Changed to Lee So-young, co-researcher -> Hyeon Jeong Lee, co-researcher</td></tr>
</table>

v 13.0

Item	Details / page	change Jeon	change after	note
Research outline	statistical Analysis method/3p	early and 6 months After, simple after 1 year X-ray imaging result, clinical evaluation statistically Compare (normal distribution) curvesee Paired t-test or Wilcoxon signed rank test compare). Also experimental group control between make the difference independent t-test or By Mann Whitney U test verify at this time The significance level is 0.05 as set	early and simple one year later X-ray filming result, clinical evaluation statistical(Paired t-test or Wilcoxon compared with signed rank test). In addition, the initial, 6 months, and 1 year Differences in change values for plain X-ray results and clinical evaluation statistically Compare. repeat measured on the value about experimental group contrastLinear mixed model or Generalized estimating between groups with equation (GEE) verify P-values are < 0.05 Work case significant to the level judge	Revised after statistical advice.
3. Principal Investigator and collaborator statement and title	Collaborator/4p	Name Location Institution Name Major Position Contact Joonyoung J a n g , Seoul National University Bundang Hospital Department of Rehabilitation Medicine full-time doctor 010-3487-7667	Name Location Institution Name Major Position Contact Jiwoon Lim Seoul National University Bundang Hospital Department of Rehabilitation Medicine full-time doctor 010-9699-6505	Researcher resignation and employment
	Researcher/4p	Researcher: S e o n C h o for clinical trials Medical Equipment manager: usually inside the park Medical Equipment manager medical device in charge be it, copy In the study to the patient according to dimension produce after so provide, Seungeun Lee researcher in charge	Researcher: Seon Cho for clinical trials Medical Equipment manager: usually inside the park Medical Equipment managermedical device in charge be it, copy In the study to the patient according todimension produce after so provide, Seon Cho esearcher in charge	Researcher resignation
12. Concrete Research method	manifold data minute three Method/11p	all study subjects registration is gun 68 people, not only institutions not Jeju National University Hospital, Pusan National University Hospital, Bundang Jesaeng Hospital,At Korea University Hospital, the study subjects were also competitively recruit of the study subject the random assignment copy institutional randomization person in charge in charge, in each institution study subjects to enroll when sequentially randomization number by giving experimental group control decide	all study subjects registration is gun 68 people, not only the institution Jeju National University Hospital, Pusan National University Hospital, Bundang Jesaeng Hospital, Korea University Hospital study subjects competitively recruit Randomization of study subjects is institutional randomization person in charge responsible, each Institutions to enroll study subjects at the time Sequentiallyrandomization number by giving the experimental group and Set a control group. Also, For the group to which the study subjects were assigned, information has been institutional random Assignment manager, each institutional research	Blinding was specified to reduce analysis bias, and the number of analyzers increased from 2 to 3.

			in charge researcher only the therapist know there is, study subjects, independent evaluator and statistics the analyst blindfolded is maintained in analysis effectiveness anti-bias for of this clinical trial Principal Investigator and each institutional The lead researcher is the patient's screening, allotment a series of in the process Not at all not involved not as do.<br>Screening and follow-up city X-ray video all keep, blindfolded became 3 phosphorous to the rater blinding became X-ray video by passing analysis to carry out do.	
17. Effect sEvaluation standard, Evaluation meth od and interp retation Method (statistical analysis method , etc.)	19p	Previous Contents none	every X- ray of reading is blinded 3 specialists and average values are used.	Blinding was specified to reduce analysis bias, and the number of analyzers increased from 2 to 3.
	data analyze and statistics analyze Method/19p	1. Primary efficacy endpoint: hip dislocation through radiographic evaluation (%)<br>scoliosis, pain index, and hip joint protection through plain radiograph wearable<br>Functional changes such as satisfaction with medical devices, hip and knee joint range of motion, discomfort when caring for a patient, and changes in the patient's overall quality of life are identified.<br>The initial and 1-year plain X-ray results were compared statistically (by Wilcoxon matched pairs signed rank test). The difference in change values is verified by Mann Whitney U test between the experimental group and the control group. If -values is < 0.05, it is judged to be a significant level. Dropouts are analyzed for all data including in statistical processing (ITT) through safety and efficacy evaluation, unless there is a justifiable reason or evidence, and PP analyzes by excluding dropouts and patients with data omissions.	1. primary effectiveness evaluation variable: radiation evaluation through hip jointdislocation (%)<br>2. secondary effectiveness evaluation variable: simple radiation check through scoliosisDegree, ache Indices, hip joint protect wearable of medical devices satisfaction,<br>hip class knee of joint range of motion, the patient take care at the time discomfort, patient'soverall of life vaginal change Such as functional change find out.<br>early and one year later simple X-ray filming result, clinical evaluation statistically Compare (normality on black according to Paired t-test or Wilcoxon signed rank test as compare). Also, early and 6 months After, 1 year after simplicity X-ray results and clinical for evaluation change of value make the difference statistically Compare. repeat measured on the value about between the experimental group and the control group linear mixed model or Generalized estimating with equation (GEE) verify P-values go < 0.05 Work case significant to the level judge<br>midway dropped out sleeping proper Reason or grounded Do not have one safety,effectiveness evaluation through statistics to processing	3.

			Including (ITT) every data Target Analyze. FAS to the military about missing value occurred in caseMissing Completely at Random (MCAR) home to be satisfied difficult Because subtract analyze, pp Is midway dropout, data omission there is the patient subtract Analyze.	
20. Materials and stability monitoring plan (DSMP)	monitoring officer /22p	(1) data and safety monitoring Responsible for: Beom Jae-wonlead researcher, data and safety monitoring manager: Seungeun Lee	(1) data and safety monitoring Responsible for: Beom Jae-won responsibilityresearcher, data and safety monitoring manager: Seon Cho	(2)
21. study subjectsof safety protection for Measures	the study subjectof Course/23p	• to the study subjects explain consent to acquire researcher: Jaewon Beom , Joonyoung Jang, Hyun-Jeong Lee, Jee Hyun Suh, Bo Ryun Kim , Yong Beom Shin<br>• consent to provide ruler: study subjects and deputy<br>• Waiting between study description process and consent acquisition process hour: 3 days Within<br>• minimize the possibility of coercion or undue	• to the study subjects explain consent to acquire researcher : Jae-Won Beom , Im Ji-woon , Hyun-Jeong Lee, Jee Hyun Suh, Bo Ryun Kim , Yong Beom Shin<br>• consent to provide ruler: study subjects and deputy<br>• Waiting time between study description process and consent acquisition process: 3 days Within<br>• minimize the possibility of coercion or undue influence; Way :	•

		influence; Way: coercive possibility or undue Affect to minimize for of the study subject research Participation Whether the decision is voluntary will, research take part reject or research term middle whenever freely take part to stop Number and by teeth Because next in treatment any disadvantage Not at all take it won't will, whenever Participation in research consent to withdraw Number that there is inform<br>research Explanation process and agreement acquisition In progress researcher using language : Korean<br>• research subject or his/her representative can understandlanguage: Korean<br>• study subjects or to the agent provided information and<br>agreement Format: consent and manual offer	To minimize the possibility of coercion or undue influence of the study subject research Participation Whether the decision is voluntary will, research take part reject or research term middlewhenever freely take part to stop Number and by teeth Because on the next treatment any no disadvantage at all take it won't and that consent to participate in the study may be withdrawn at any time. inform<br>• research Explanation process and agreement acquisition In progress researcher using language: Korean<br>• study subjects or agent understand Number there is language: Korean<br>study subjects or to the agent provided information and agreement Format: consent and manual offer	

This supplement contains the following items:

1. Statistical Analysis Plan

Version 2.0 (Page 2)

Version 13.0 (Page 8)

Summary of Changes from Version 2.0 to Version 13.0 (Page 15)

Statistical Analysis Plan, Version 2.0

1.0 Introduction

This Statistical Analysis Plan (SAP) describes the statistical analyses planned to address the objectives of the clinical trial. It details the analyses that will be performed to accomplish these objectives. This SAP defines variables and identifies methods and algorithms used to populate the tables, figures, and listings that are included in reports for this study.

2.0 Study Design

2.1 Objectives

2.1.1 Primary Objectives

The primary purpose of this study is to investigate the purpose of our study was to investigate the efficacy of newly designed hip protection brace applying biomechanical factor for preventing progressive hip displacement in patients with severe CP in a single-blinded randomized clinical trial (RCT).

2.1.2 Secondary Objectives

The second purpose is to investigate the safety of hip protection brace.

2.2 Study Hypotheses

Our hypothesis is that hip protection brace can stabilize the hip joints, reduce the activation of hip adductors, and assist the protective function of ligaments and muscles around hip joints, thereby decreasing the progression of hip dislocation.

2.3 Sample Size Determination

2.3.1 Statistical Hypotheses

the null hypothesis is as following.
H0= There is no meaningful relationship in migration index (MI) between the two groups. (H0: Ut=Uc).
H1 = There is significant differences in MI between the two groups. (H1:Ut≠Uc).
Ut = MI (Post, test group) - MI (Pre, test group), Uc= MI (Post, control group) - MI (Pre, control group).

2.3.2 Sample Size Determination

A. The calculation of the sample size is based on the previous epidemiologic study (Kim IS, Park D, Ko JY, Ryu JS. Are Seating Systems With a Medial Knee Support Really Helpful for Hip Displacement in Children With Spastic Cerebral Palsy GMFCS IV and V? *Arch Phys Med Rehabil* 2019; **100**(2): 247-53.).
B. In the previous study of this researcher, the MI of GMFCS IV and V patients before wearing auxiliary equipment was measured to be 26.89±17.10%
C. In this study, the MI of the experimental group was set to 16.134±17.1 and 13.445±17.1, respectively, assuming that the MI decreased by 40% for GMFCS IV and 50% for GMFCS V when wearable auxiliary

medical devices were worn.

D. We used (G-power 3.1.9.2) for two-sample T-Test assuming equal variance.

E. With an α of less than 0.05 in the two-tailed tests and a power of 80%, the target sample size of each group is 11 patients. Considering a dropout rate of 5%, the total sample size was calculated as 11 each in the experimental group and control group for GMFCS IV, and 8 each in the experimental group and control group for GMFCS V. Considering the dropout rate of 10%, a total of 42 experimental and control groups will be recruited.

F. This clinical trial compares the post-treatment changes between the study and the control groups. This study was the superiority trial to show the significant improvement of the MI in the study group than the control group.

2.4 Blinding

This clinical trial is conducted with single blinded (covering the eyes of the evaluators) to minimize bias. For blinding of the evaluators, all evaluators are blinded to the group allocation and blinded the clinical information.

Only the random assignment officer and the therapist (the researcher) who conducted the intervention are aware of the information about the group allocation. But the independent evaluators are kept blinded. After all test results are saved as images or entered into the database and database locking, the person in charge can know the randomly assigned results.

2.5 Assessment

The assessment will be performed by two independent physiatrists who have certificate.

2.6 Randomization and Enrollment

In order to prevent an imbalance in the number of study subjects between the experimental group and the control group, a block randomization method is adopted. Randomization was performed using Microsoft Excel program. 24 GMFCS IV and 18 GMFCS V subjects were randomly assigned to two groups with a block size of 4 and an allocation ratio of 1:1. In the header of cells A1, B1, C1, and D1 of the Excel spreadsheet, enter Sequence, Random number, Block, and Group, respectively. ① Enter the numbers from 1 to 24 and 1 to 18 in cell A, which is the Sequence column, in order, and ② enter the function = Rand() in B of the Random number column to assign a random function. ③ In block column C, repeat the numbers 1 to 6 and 1 to 4 in order. At this time, the numbers 1 to 4 or 6 are repeated 4 times, which means that 24 and 18 study subjects were assigned a block size of 4. ④ In the Group column, input the experimental group and the control group, respectively, followed by 12 and 9 people. Then, drag from B1 to D1 to select it, and select Home -> Sort & Filter -> Filter. When there is a scroll bar in each cell, click

the scroll bar in C1 to select ascending sort, and then click the scroll bar in C1 to sort in ascending order to complete the randomization table (Kang, 2017). A random number table is created, and a new random function is generated at the start of the study. At this time, the random number on the sheet may change and the assignment may be changed, so copy or capture the value and keep it.

2.7 Outcomes

2.7.1 Primary Outcome

Hip dislocation by radiographic evaluation (%).

2.7.2 Secondary Outcomes

· Functional changes such as the degree of scoliosis, pain index, satisfaction with wearable medical devices for hip joint protection, hip and knee joint range of motion, discomfort in caring for patients, and changes in the overall quality of life of patients through simple radiographic examination.

A. Joint range of motion

Measure the joint range of motion of the patient's hip and knee using a goniometer.

Ⅰ. abduction with hip at 90’ flexion

Ⅱ. Hip abduction with hip at 0’ flexion

Ⅲ. Hip adduction with hip at 0’ flexion

Ⅳ. Hip flexion contracture (Thomas test)

Ⅴ. Popliteal angle

B. Questionnaire evaluation

I. Pain index (Visual analog scale): In the case of children with cerebral palsy GMFCS IV and V, communication is difficult, so the patient's hip pain felt by the guardian is evaluated.

C. Likert scale

Satisfaction evaluation of wearable medical device for hip joint protection

Example of this study questionnaire: Very satisfied - Satisfied - Moderate - Somewhat dissatisfied - Very dissatisfied

D. Child Health Index of Life with Disabilities (CPCHILD)

This tool consists of 9 items as a tool to evaluate the quality of life of patients with severe cerebral palsy. The guardian evaluates, and each item is scored on a scale of 0 to 5, and each item is as follows. Investigate hip contracture-related items among CPCHILDs, such as daily activities, posture/movement, comfort and emotion, the child's overall quality of life, and the importance of items related to the child's quality of life

2.8 Study Design

2.8.1 Overall Design

Multicenter, prospective, parallel, double blinded, randomized clinical trial.

2.8.2 Study Scheme

- Patients diagnosed with severe cerebral palsy were included in the study.
- Assignment to experimental group and control group when consent to the study
- Radiologic and clinical evaluation were performed to evaluate hip dislocation.
- The experimental group manufactured and applied hip joint wearable medical devices and continued the existing rehabilitation treatment.
- The control group continued the existing rehabilitation treatment.
- After 6 months and 1-year, clinical evaluation and radiological evaluation were performed to determine effectiveness.
-

2.9 Study Population

2.9.1 Inclusion Criteria

Subjects must satisfy all of the following criteria to be included in the study

I. age 1 to 15 years

II. Gross Motor Function Classification System (GMFCS) IV and V

III. quadriplegia or diplegia for more than 6 months

IV. patients who have provided written consent with permission of the child and caregiver

2.9.2 Exclusion Criteria

I. In the event that the study was discontinued at the request of the subject or his/her representative

II. In case of concurrent use of surgery, drug, or medical device that may affect clinical trial results

III. If clinical trials are not properly conducted

IV. In case the initial and late evaluation cannot be performed due to the absence of the subject

V. Withdrawal of consent at the request of the subject or his/her representative

VI. In case tracking is not possible due to non-scheduled visit

VII. In case the subject dies due to reasons not related to the clinical trial

VIII. When the investigator determines that the suspension of the test is beneficial to the subject

IX. If the other investigator determines that there is a problem with the clinical trial

3.0 Data Safety Monitoring Plan

3.1 Data Safety Monitoring Plan

3.1.1 Responsibility of Data and Safety Monitoring: Ju Seok Ryu, The Principal Investigator

3.1.2 Practice of Data and Safety Monitoring: Ah-reum Shin, The Sub- Investigator

Risk occurrence of this study is low since only one evaluation of X-ray and brace(elastic

bandage) is performed. This study is corresponded to a low risk study. Measure pain and discomfort before and after the test. To decide continue, change or discontinue of the study, use Numerical Rating Scale (NRS) as a key validation variable.

If the pain and the discomfort last 3 days after the test, perform additional test and confirm safety. The test fee should be paid by the insurance that have signed up for. To manage this, Ju Seok Ryu professor is appointed as the monitoring manager and the principal investigator and the sub-investigator perform monitoring every six months. In the adverse event during the study, if the case is a serious adverse effect, report it to the IRB immediately and if the case is a minor adverse effect, the principal investigator and the sub-investigator perform monitoring first and report it to the IRB.

3.1.3 Notifying IRB, principal investigator and Regulatory Authorities

I. Adverse drug effect/adverse medical device effect report (fatal/life-threatening): Within 7 days (Initial report), within 8 days (tracking report)

II. Adverse drug effect/adverse medical device effect report (not fatal/life-threatening): Within 15 days

III. Safety information report: At least once every 6 months

IV. Report of unexpected adverse reactions: Within 15 days

3.1.4 Criteria for discontinuance of the study

I. Discontinue the study when the target study data is obtained.

II. If the number of study subjects could not be recruited within the IRB approval period, determine the discontinuation or alternation of the study.

III. If the study is discontinued, the subjects follow the treatment procedures of the correspond hospital.

4.0 General statistical considering

4.1 This clinical trial uses a two-sided hypothesis for the test of difference in effectiveness. It is defined as a two-sided hypothesis, as stated in the section "Sample size calculation". (H0: Ut=Uc, H1: Ut≠U).

4.2 The statistical method is to test normality using the Kolmogorov-Smirnov test, then test parametric or Wilcoxon crank-sum test (nonparametric test) depending on the normal distribution.

4.3 The number of observations, mean, standard deviation, median, minimum, and maximum values are presented for continuous variables, and frequency and percentage are presented for categorical data.

4.4 If a missing value occurs during validity analysis or a missing value occurs due to the elimination of the subject before the clinical trial is completed, this study measures the validity evaluation only once in the early and late period, and analyzes only the observed data (observed processes) without any correction to the missing values.

4.5 However, other safety assessment and other data are analyzed using data that do not replace missing values.

4.6 Assessment of effectiveness

This clinical trial evaluates the p-value v 0.05 as statistically significant results after conducting a two-sided test with either a T-test or a Wilcoxon rank-sum test.

4.7 Definition of the Evaluation and Analysis Group

The main analysis group of this clinical trial is Full analysis set, the additional analysis is for per-protocol set, and the data on stability is analyzed for safety.

Statistical Analysis Plan, Version 13.0

1.0 Introduction

This Statistical Analysis Plan (SAP) describes the statistical analyses planned to address the objectives of the clinical trial. It details the analyses that will be performed to accomplish these objectives. This SAP defines variables and identifies methods and algorithms used to populate the tables, figures, and listings that are included in reports for this study.

2.0 Study Design

2.1 Objectives

2.1.1 Primary Objectives

The primary purpose of this study is to investigate the purpose of our study was to investigate the efficacy of newly designed hip protection brace applying biomechanical factor for preventing progressive hip displacement in patients with severe CP in a single-blinded randomized clinical trial (RCT).

2.1.2 Secondary Objectives

The second purpose is to investigate the safety of hip protection brace.

2.2 Study Hypotheses

Our hypothesis is that hip protection brace can stabilize the hip joints, reduce the activation of hip adductors, and assist the protective function of ligaments and muscles around hip joints, thereby decreasing the progression of hip dislocation.

2.3 Sample Size Determination

2.3.1 Statistical Hypotheses

the null hypothesis is as following.

H0= There is no meaningful relationship in migration index (MI) between the two groups. (H0: Ut=Uc).

H1 = There is significant differences in MI between the two groups. (H1:Ut≠Uc).

Ut = MI (Post, test group) - MI (Pre, test group), Uc= MI (Post, control group) - MI (Pre, control group).

2.3.2 Sample Size Determination

A. The calculation of the sample size is based on the previous epidemiologic study (Kim IS, Park D, Ko JY, Ryu JS. Are Seating Systems With a Medial Knee Support Really Helpful for Hip Displacement in Children With Spastic Cerebral Palsy GMFCS IV and V? *Arch Phys Med Rehabil* 2019; **100**(2): 247-53.).

B. In this study, the annual progression of MI in patients with severe cerebral palsy (GMFCS IV and V) was measured to be 7.83 ± 8.73 %. Assuming the MI decreased by 80 % applying Hip protection brace, the MI of the study group was set to 1.566 ±

8.73 %.

C. We used PASS 15.0 for two-sample T-Test assuming equal variance.

D. With an α of less than 0.05 in the two-tailed tests and a power of 80%, the target sample size of each group is 32 patients.

E. Considering a dropout rate of 5 %, the final sample size was determined to be 68 patients per group.

F. This clinical trial compares the post-treatment changes between the study and the control groups. This study was the superiority trial to show the significant improvement of the MI in the study group than the control group.

1.1 Blinding

This clinical trial is conducted with single blinded (covering the eyes of the evaluators) to minimize bias. For blinding of the evaluators, all evaluators are blinded to the group allocation and blinded the clinical information.

Only the random assignment officer and the therapist (the researcher) who conducted the intervention are aware of the information about the group allocation. But the independent evaluators are kept blinded. After all test results are saved as images or entered into the database and database locking, the person in charge can know the randomly assigned results.

1.2 Assessment

The assessment will be performed by three independent physiatrists who have certificate.

1.3 Randomization and Enrollment

In order to prevent an imbalance in the number of study subjects between the experimental group and the control group, a block randomization method is adopted. Randomization was performed using Microsoft Excel program. 24 GMFCS IV and 18 GMFCS V subjects were randomly assigned to two groups with a block size of 4 and an allocation ratio of 1:1. In the header of cells A1, B1, C1, and D1 of the Excel spreadsheet, enter Sequence, Random number, Block, and Group, respectively. ① Enter the numbers from 1 to 24 and 1 to 18 in cell A, which is the Sequence column, in order, and ② enter the function = Rand() in B of the Random number column to assign a random function. ③ In block column C, repeat the numbers 1 to 6 and 1 to 4 in order. At this time, the numbers 1 to 4 or 6 are repeated 4 times, which means that 24 and 18 study subjects were assigned a block size of 4. ④ In the Group column, input the experimental group and the control group, respectively, followed by 12 and 9 people. Then, drag from B1

to D1 to select it, and select Home -> Sort & Filter -> Filter. When there is a scroll bar in each cell, click the scroll bar in C1 to select ascending sort, and then click the scroll bar in C1 to sort in ascending order to complete the randomization table (Kang, 2017). A random number table is created, and a new random function is generated at the start of the study. At this time, the random number on the sheet may change and the assignment may be changed, so copy or capture the value and keep it.

Enrollment : competitive enrollment

1.4 Outcomes

1.4.1 Primary Outcome

Hip dislocation by radiographic evaluation (%).

1.4.2 Secondary Outcomes

· Functional changes such as the degree of scoliosis, pain index, satisfaction with wearable medical devices for hip joint protection, hip and knee joint range of motion, discomfort in caring for patients, and changes in the overall quality of life of patients through simple radiographic examination.

A. Joint range of motion

Measure the joint range of motion of the patient's hip and knee using a goniometer.

VI. abduction with hip at 90’ flexion

VII. Hip abduction with hip at 0’ flexion

VIII. Hip adduction with hip at 0’ flexion

IX. Hip flexion contracture (Thomas test)

X. Popliteal angle

B. Femur anteversion

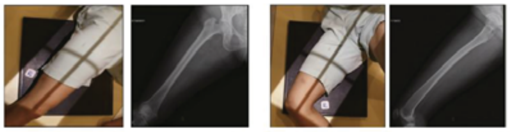

Fig. 1. Anterior–posterior (AP) and lateral (LAT) imaging protocols. In the LAT view, the pelvis is rotated slightly posteriorly to prevent overlap with the femur head.

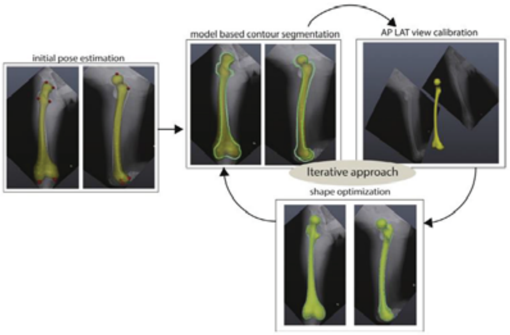

Fig 2. System overview: the anterior–posterior (AP) and lateral (LAT) views are initially pose-estimated using user inputted six points and its 3D correspondences. The image contour is segmented based on the projected contour of the 3D femur. The AP and LAT views are calibrated more accurately using contour correspondences. The shape is optimized so that it projected contour to match the segmented contour. These steps are iteratively performed until convergence.

In the case of femur anteversion, CT is generally taken, but the radiation dose is high, so we decided to use a method to measure femur anteversion using simple radiographic examinations such as femur AP and femur lateral x-ray. (K. Youn et al) Measured at initial, 6 months, and 12 months.

C. Questionnaire evaluation

I. Pain index (Visual analog scale): In the case of children with cerebral palsy GMFCS IV and V, communication is difficult, so the patient's hip pain felt by the guardian is evaluated.

D. Likert scale

Satisfaction evaluation of wearable medical device for hip joint protection

Example of this study questionnaire: Very satisfied - Satisfied - Moderate - Somewhat dissatisfied - Very dissatisfied

E. Child Health Index of Life with Disabilities (CPCHILD)

This tool consists of 9 items as a tool to evaluate the quality of life of patients with severe cerebral palsy. The guardian evaluates, and each item is scored on a scale of 0 to 5, and each item is as follows. Investigate hip contracture-related items among CPCHILDs, such as daily activities, posture/movement, comfort and emotion, the child's overall quality of life, and the importance of items related to the child's quality of life

1.5 Study Design

1.5.1 Overall Design

Multicenter, prospective, parallel, double blinded, randomized clinical trial.

1.5.2 Study Scheme

- Patients diagnosed with severe cerebral palsy were included in the study.
- Assignment to experimental group and control group when consent to the study
- Radiologic and clinical evaluation were performed to evaluate hip dislocation.
- The experimental group manufactured and applied hip joint wearable medical devices and continued the existing rehabilitation treatment.
- The control group continued the existing rehabilitation treatment.
- After 6 months and 1-year, clinical evaluation and radiological evaluation were performed to determine effectiveness.
-

1.6 Study Population

1.6.1 Inclusion Criteria

Subjects must satisfy all of the following criteria to be included in the study

I. age 1 to 15 years

II. Gross Motor Function Classification System (GMFCS) IV and V

III. quadriplegia or diplegia for more than 6 months

IV. patients who have provided written consent with permission of the child and caregiver

1.6.2 Exclusion Criteria

I. In the event that the study was discontinued at the request of the subject or his/her representative

II. In case of concurrent use of surgery, drug, or medical device that may affect clinical trial results

III. If clinical trials are not properly conducted

IV. In case the initial and late evaluation cannot be performed due to the absence of the subject

V. Withdrawal of consent at the request of the subject or his/her representative

VI. In case tracking is not possible due to non-scheduled visit

VII. In case the subject dies due to reasons not related to the clinical trial

VIII. When the investigator determines that the suspension of the test is beneficial to the subject

IX. If the other investigator determines that there is a problem with the clinical trial

1.7 Data Analysis and Statistical Analysis Methods

- The clinical evaluation is statistically compared between the initial and simple X-ray results after 1 year (compare with paired t-test or Wilcoxon signed rank test according to normality test).
- In addition, the difference in the change values for the initial, 6 months, and 1 year after simple X-ray imaging and clinical evaluation is statistically compared.
- Repeatedly measured values are verified with a linear mixed model or Generalized estimating equation (GEE) between the experimental group and the control group.
- If the P-values are < 0.05, it is judged to be a significant level.
- All data, including those included in statistical processing (ITT) through safety and efficacy evaluation, will be analyzed for those who are dropped out unless there is a valid reason or justification.
- In the case of missing values in the FAS group, it is difficult to satisfy the assumption of Missing Completely at Random (MCAR), so it is excluded and analyzed.

2.0 Data Safety Monitoring Plan

2.1 Data Safety Monitoring Plan

3.1.1 Responsibility of Data and Safety Monitoring: Ju Seok Ryu, The Principal Investigator

3.1.5 Practice of Data and Safety Monitoring: Seon Cho, The Sub- Investigator

Risk occurrence of this study is low since only one evaluation of X-ray and brace(elastic bandage) is performed. This study is corresponded to a low risk study. Measure pain and discomfort before and after the test. To decide continue, change or discontinue of the

study, use Numerical Rating Scale (NRS) as a key validation variable.

If the pain and the discomfort last 3 days after the test, perform additional test and confirm safety. The test fee should be paid by the insurance that have signed up for. To manage this, Ju Seok Ryu professor is appointed as the monitoring manager and the principal investigator and the sub-investigator perform monitoring every six months. In the adverse event during the study, if the case is a serious adverse effect, report it to the IRB immediately and if the case is a minor adverse effect, the principal investigator and the sub-investigator perform monitoring first and report it to the IRB.

3.1.6 Notifying IRB, principal investigator and Regulatory Authorities

I. Adverse drug effect/adverse medical device effect report (fatal/life-threatening): Within 7 days (Initial report), within 8 days (tracking report)

II. Adverse drug effect/adverse medical device effect report (not fatal/life-threatening): Within 15 days

III. Safety information report: At least once every 6 months

IV. Report of unexpected adverse reactions: Within 15 days

3.1.7 Criteria for discontinuance of the study

I. Discontinue the study when the target study data is obtained.

II. If the number of study subjects could not be recruited within the IRB approval period, determine the discontinuation or alternation of the study.

III. If the study is discontinued, the subjects follow the treatment procedures of the correspond hospital.

5.0 General statistical considering

4.8 This clinical trial uses a two-sided hypothesis for the test of difference in effectiveness. It is defined as a two-sided hypothesis, as stated in the section "Sample size calculation". (H0: Ut=Uc, H1: Ut≠U).

4.9 The statistical method is to test normality using the Kolmogorov-Smirnov test, then test parametric or Wilcoxon crank-sum test (nonparametric test) depending on the normal distribution.

4.10 The number of observations, mean, standard deviation, median, minimum,

and maximum values are presented for continuous variables, and frequency and percentage are presented for categorical data.

4.11 If a missing value occurs during validity analysis or a missing value occurs due to the elimination of the subject before the clinical trial is completed, this study measures the validity evaluation only once in the early and late period, and analyzes only the observed data (observed processes) without any correction to the missing values.

4.12 However, other safety assessment and other data are analyzed using data that do not replace missing values.

4.13 Assessment of effectiveness

Repeatedly measured values are verified with a linear mixed model or Generalized estimating equation (GEE) between the experimental group and the control group. If the P-values are < 0.05, it is judged to be a significant level.

4.14 Definition of the Evaluation and Analysis Group

The main analysis group of this clinical trial is Full analysis set, the additional analysis is for per-protocol set, and the data on stability is analyzed for safety.

List of Amentment

Amendment is on the underline.

Section	Detailed item /page	Version 2.0	Version 13.0	Reason for Amendment
SAP	2.3.2 A. Sample Size Calculation, p28	In this study, the MI of the experimental group was set to 16.134±17.1 and 13.445±17.1, respectively, assuming that the MI decreased by 40% for GMFCS IV and 50% for GMFCS V when wearable auxiliary medical devices were worn.<br>We used (G-power 3.1.9.2) for two-sample T-Test assuming equal variance.<br>With an α of less than 0.05 in the two-tailed tests and a power of 80%, the target sample size of each group is 11 patients. Considering a dropout rate of 5%, the total sample size was calculated as 11 each in the experimental group and control group for GMFCS IV, and 8 each in the experimental group and control group for GMFCS V. Considering the dropout rate of 10%, a total of 42 experimental and control groups will be recruited.	A. In this study, the annual progression of MI in patients with severe cerebral palsy (GMFCS IV and V) was measured to be 7.83 ± 8.73 %. Assuming the MI decreased by 80 % applying Hip protection brace, the MI of the study group was set to 1.566 ± 8.73 %.<br>B. We used PASS 15.0 for two-sample T-Test assuming equal variance.<br>C. With an α of less than 0.05 in the two-tailed tests and a power of 80%, the target sample size of each group is 32 patients.<br>D. Considering a dropout rate of 5 %, the final sample size was determined to be 68 patients per group.	Recommended by Statistician at IRB meeting

SAP	2.5. Assessment	The assessment will be performed by two independent physiatrists who ave certificate.	The assessment will be performed by three independent physiatrists who have certificate.	To reduce bias
SAP	2.7 Outcome<br>2.7.2 Secondary outcome<br>B. Femur anteversion		**B. Femur anteversion**<br>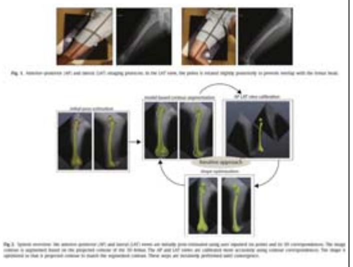	Added analysis of femur anteversion to determine the effect of hip protection brace on coxa valga.
SAP	**Data Analysis and Statistical Analysis Methods**		The clinical evaluation is statistically compared between the initial and simple X-ray results after 1 year (compare with paired t-test or Wilcoxon signed rank test according to normality test).	Changed after Statistical consultation

			In addition, the difference in the change values for the initial, 6 months, and 1 year after simple X-ray imaging and clinical evaluation is statistically compared. Repeatedly measured values are verified with a linear mixed model or Generalized estimating equation (GEE) between the experimental group and the control group. If the P-values are < 0.05, it is judged to be a significant level.<br>All data, including those included in statistical processing (ITT) through safety and efficacy evaluation, will be analyzed for those who are dropped out unless there is a valid reason or justification.<br>In the case of missing values in the FAS group, it is difficult to satisfy the assumption of Missing Completely at Random (MCAR), so it is excluded and analyzed.	
SAP	3.1.2 Practice of Data and Safety Monitoring	Ah-reum Shin	: Seon Cho	Changed by the resignation of the researcher.

SAP	4.6 Assessment of effectiveness	This clinical trial evaluates the p-value v 0.05 as statistically significant results after conducting a two-sided test with either a T-test or a Wilcoxon rank-sum test.	Repeatedly measured values are verified with a linear mixed model or Generalized estimating equation (GEE) between the experimental group and the control group. If the P-values are < 0.05, it is judged to be a significant level.	Changed after Statistical consultation
