# Supplementary material for: Efficacy of a Hip Brace for Hip Displacement in Children With Cerebral Palsy: A Randomized Clinical Trial
Source: JAMA Netw Open. 2022 Nov 4;5(11):e2240383. doi: 10.1001/jamanetworkopen.2022.40383 (PMC9636519; doi:10.1001/jamanetworkopen.2022.40383)
Supplement: Supplement 2. — eFigure. Clothing Pressure Measurements eTable. Results of the Linear Mixed Model [file jamanetwopen-e2240383-s002.pdf]

Supplemental Online Content

Kim BR, Yoon JA, Han HJ, et al. Efficacy of a hip brace for hip displacement in children with cerebral palsy: a randomized clinical trial. *JAMA Netw Open*. 2022;5(11):e2240383. doi:10.1001/jamanetworkopen.2022.40383

eFigure. Clothing Pressure Measurements

eTable. Results of the Linear Mixed Model

This supplemental material has been provided by the authors to give readers additional information about their work.

© 2022 Kim BR et al. *JAMA Network Open.*

eFigure 1. The clothing pressure measurement

9

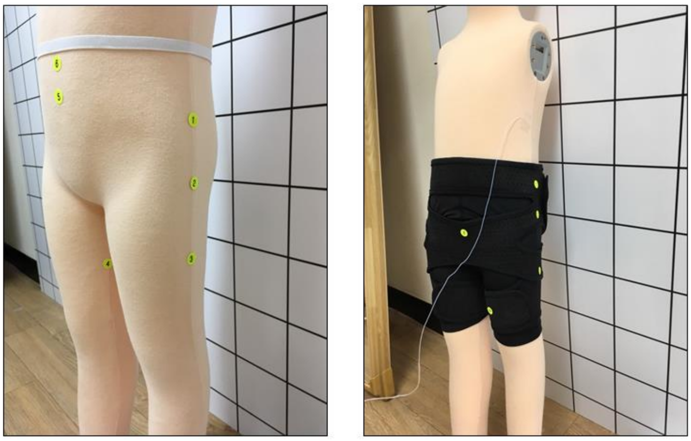

Location Number	Location	Pressure class
1	Upper straps	III
2	Lower straps	II
3	Lateral thigh	IV
4	Medial thigh	IV
5	Below umbilicus	I
6	Above umbilicus	I
7	Buttock	IV

European Committee for Standardization, 2001

© 2022 Kim BR et al. *JAMA Network Open.*

eTable 1. The results of a linear mixed model

<Type III test>

row.name	Sum Sq	Mean Sq	NumDF	DenDF	F value	Pr(>F)<br>P value
Age (yr)	68.37907	68.37907	1	65.37849	2.307281	0.1336
baseline MI	150448.74	150448.74	1	881.35367	5076.5164	0.0000
Group 1 vs 2	1567.6234	1567.6234	1	67.2872	52.895532	0.0000
Time	1272.1703	636.08513	2	992.76339	21.463102	0.0000
**Group:Time**	7140.8174	3570.4087	2	992.75918	120.47451	**0.0000**

<with interaction>

row.name	Estimate	SE	df	t.value	p.value
(Intercept)	2.609	1.211	86.774	2.155	0.0340
Age (yr)	-0.293	0.193	65.378	-1.519	0.1336
baseline MI	0.959	0.013	881.354	71.250	0.0000
Group 1 vs 2	0.481	1.015	95.702	0.474	0.6365
Time 6mo vs baseline	5.939	0.554	974.877	10.712	0.0000
Time 12mo vs baseline	9.396	0.574	981.527	16.359	0.0000
Group 1:Time 6mo	-8.642	0.830	998.499	-10.417	0.0000
Group 1:Time 12mo	-13.134	0.874	1003.775	-15.027	0.0000

<without interaction>

row.name	Estimate	SE	df	t.value	p.value
(Intercept)	6.094	1.283	86.213	4.748	0.0000
Age (yr)	-0.310	0.199	63.694	-1.561	0.1235
baseline MI	0.947	0.020	129.294	47.841	0.0000
Group 1 vs 2	-5.463	0.958	65.396	-5.701	0.0000
Time 6mo vs baseline	2.034	0.439	935.123	4.633	0.0000
Time 12mo vs baseline	3.681	0.461	940.495	7.990	0.0000

© 2022 Kim BR et al. *JAMA Network Open.*
