# Supplementary material for: Efficacy of a Hip Brace for Hip Displacement in Children With Cerebral Palsy: A Randomized Clinical Trial
Source: JAMA Netw Open. 2022 Nov 4;5(11):e2240383. doi: 10.1001/jamanetworkopen.2022.40383 (PMC9636519; doi:10.1001/jamanetworkopen.2022.40383)
Supplement: Supplement 3. — Data Sharing Statement [file jamanetwopen-e2240383-s003.pdf]

Data Sharing Statement

Kim. Efficacy of a Hip Brace for Hip Displacement in Children With Cerebral Palsy. *JAMA Netw Open*. Published November 04, 2022. doi:10.1001/jamanetworkopen.2022.40383

Data
Data available: Yes
Data types: Deidentified participant data
How to access data: request for data must be sent to an individual. email : jseok337@daum.net
When available: With publication

Supporting Documents
Document types: Statistical/analytic code, Informed consent form
How to access documents: jseok337@daum.net
When available: With publication

Additional Information
Who can access the data: researchers whose proposed use of the data has been approved
Types of analyses: for a specified purpose
Mechanisms of data availability: with investigator support after approval of a proposal
